# Ferrite Nanoparticles-Based Reactive Oxygen Species-Mediated Cancer Therapy

**DOI:** 10.3389/fchem.2021.651053

**Published:** 2021-04-27

**Authors:** Shancheng Yu, Huan Zhang, Shiya Zhang, Mingli Zhong, Haiming Fan

**Affiliations:** ^1^School of Biomedical Engineering and Informatics, Nanjing Medical University, Nanjing, China; ^2^College of Chemistry and Materials Science, Northwest University, Xi'an, China; ^3^School of Public Health, Nanjing Medical University, Nanjing, China

**Keywords:** ferrite nanoparticles, reactive oxygen species, cancer therapy, fenton reaction, external field, cascade reaction

## Abstract

Ferrite nanoparticles have been widely used in the biomedical field (such as magnetic targeting, magnetic resonance imaging, magnetic hyperthermia, etc.) due to their appealing magnetic properties. In tumor acidic microenvironment, ferrite nanoparticles show intrinsic peroxidase-like activities, which can catalyze the Fenton reaction of hydrogen peroxide (*H*_2_*O*_2_) to produce highly toxic hydroxyl free radicals (•*OH*), causing the death of tumor cell. Recent progresses in this field have shown that the enzymatic activity of ferrite can be improved *via* converting external field energy such as alternating magnetic field and near-infrared laser into nanoscale heat to produce more •*OH*, enhancing the killing effect on tumor cells. On the other hand, combined with other nanomaterials or drugs for cascade reactions, the production of reactive oxygen species (ROS) can also be increased to obtain more efficient cancer therapy. In this review, we will discuss the current status and progress of the application of ferrite nanoparticles in ROS-mediated cancer therapy and try to provide new ideas for this area.

## Introduction

Cancer is one of the principal causes of morbidity and mortality in every country of the world. According to global cancer statistics of the World Health Organization, there were 18.1 million new cancer cases and 9.6 million cancer deaths in 2018, with the number of new cases rising 42.5% compared to that in 2008 (12.7 million) (Bray et al., 2018). In order to prevent the uncontrollable growth of tumor cells, the most conventional cancer therapeutic approaches used in clinical practice now are still surgery, chemotherapy, radiotherapy, and combination of them (Vahrmeijer et al., 2013; Barton et al., 2014; Prigerson et al., 2015; Sullivan et al., 2015; Sharma et al., 2016). However, surgery is often ineffective for advanced and metastasized cancers. Chemotherapy and radiotherapy suffer from severe side effects on account of the toxicity to normal cells and tissues. Based on the research of cancer-related biology and the development of biomedical engineering, a variety of alternative treatment strategies have been extensively studied to obtain more efficient cancer therapy, such as magnetic hyperthermia, photothermal therapy, photodynamic therapy, immunotherapy, and gene therapy (Dolmans et al., 2003; Yang et al., 2010; Kumar and Mohammad, 2011; Pardoll, 2012; Topalian et al., 2012; High and Roncarolo, 2019; Liu et al., 2019b). Most of these treatment strategies need to rely on the regulation of reactive oxygen species (ROS) to mediate tumor cell death. ROS are categorized as a class of incomplete reduction products of oxygen, mainly including superoxide anion ($O_{2}^{-}$), hydrogen peroxide (*H*_2_*O*_2_), hydroxyl radical (•*OH*), and singlet oxygen (^1^*O*_2_) (Kumari et al., 2018). Superoxide anion can be generated as a byproduct of the electron transport chain in mitochondria or through activation of nicotinamide adenine dinucleotide phosphate oxidase (NOX) and exogenous stimulation (Murphy, 2009). Superoxide dismutase can reduce superoxide to hydrogen peroxide, which can be further converted into non-oxidizing water by cytosolic antioxidant systems under the catalysis of catalase, peroxiredoxins, and glutathione peroxidase (Winston and Giulio, 1991). The balance between the production and neutralization of reactive oxygen species in normal cells is beneficial to maintaining a proper ROS concentration to regulate intracellular signaling and homeostasis (Forman et al., 2010). Reactive oxygen species at high levels can damage proteins, lipids, and DNA, resulting in mutations and carcinogenesis in normal cells (Trachootham et al., 2009). Compared with normal cells, most tumor cells metabolize in distinct pathways leading to excessive ROS production (Schumacker, 2006). Cancer cells also have a higher level of antioxidant enzymes to enable them to survive in the presence of intrinsic oxidative stress without apoptosis (Birben et al., 2012). Increasing generation, regulating the types of reactive oxygen species, and inhibiting cellular glutathione peroxidase can break the balance between the production and elimination of ROS in tumor cell and tune the function of intracellular ROS from tumor promoting toward apoptotic signaling, inducing the apoptosis and death of tumor cell for cancer therapeutics (Liou and Storz, 2010). In order to enhance the effect of ROS-mediated tumor-specific therapeutic, various drugs and nanomaterials such as doxorubicin, cisplatin, Fe_3_O_4_, gold, silver, polyoxomolybdate (POM), and molybdenum carbide have been studied for targeted delivery to tumor tissues and endocytosis by tumor cells to selectively increase the production of highly toxic ROS in tumor cells (Yanagie et al., 2006; He et al., 2012, 2016b; Maji et al., 2015; Kankala et al., 2017; Feng et al., 2019; Liu et al., 2019a; Dong et al., 2020; Maiti et al., 2020). Among these nanomaterials, ferrite nanoparticles are widely studied due to unique magnetic properties and relatively high safety to human body, especially iron oxide nanoparticles, which have been approved by the US Food and Drug Administration for clinical applications, such as iron supplement, magnetic resonance contrast agent, and drug carrier (Liu et al., 2019c). Ferrite nanomaterials are composed of main ferric oxide and one or more oxides of other metals (such as manganese, copper, nickel, cobalt, or zinc). In tumor acidic microenvironment, ferrite nanoparticles exhibit peroxidase-like activity, which can catalyze the Fenton reaction of *H*_2_*O*_2_ to produce highly toxic •*OH*, inducing the death of tumor cell (Chen et al., 2012). The peroxidase activity depends on the intrinsic properties of ferrite nanoparticles (chemical composition, crystalline phase, and particle size) and ROS-related bio-microenvironmental factors (physiological pH and buffers, biogenic reducing agents, and other organic substances). For further reading these factors in detail, an excellent review has been published by Yin and colleagues (Wu et al., 2014). In this review, we summarized the advances in the application of ferrite nanoparticles in ROS-mediated cancer therapy, and constructive perspectives were also provided.

## Ferrite-Based ROS-Mediated Cancer Therapy

**Scheme 1:**
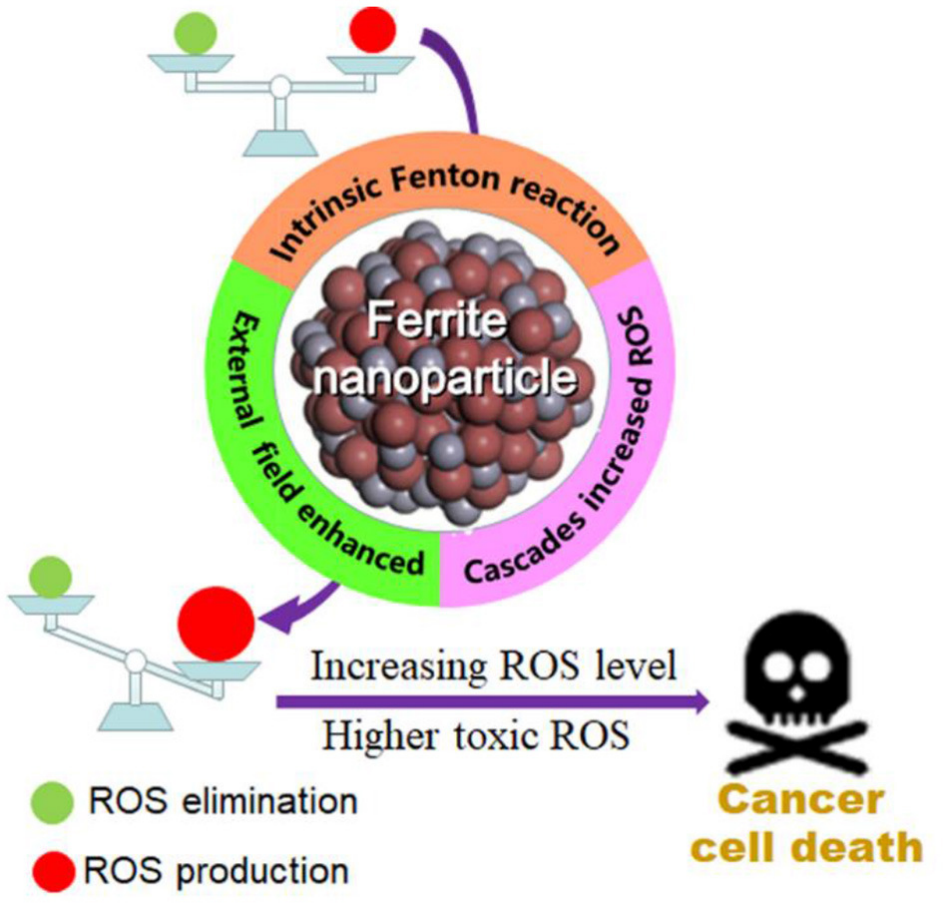
Schematic illustration of the ferrite nanoparticles-based ROS-mediated cancer therapy. Increasing generation of highly toxic ROS under the catalysis of ferrite nanoparticles can break the balance between the production and elimination of ROS based on these mechanisms: (1) intrinsic Fenton reaction catalytic activity of ferrite nanoparticles, (2) external field enhanced Fenton reaction, and (3) cascade reactions increased ROS.

In 2007, Yan et al. first discovered that Fe_3_O_4_ nanoparticles possess intrinsic peroxidase-like activity, which can catalyze the disproportionation of H_2_O_2_ to produce highly toxic •*OH* (Gao et al., 2007). Subsequently, researchers conducted extensive investigation on ferrite nanomaterials as nanoenzyme to mediate the generation of ROS for tumor treatment (Mai and Hilt, 2017). The specific mechanisms of the sufficient and highly toxic ROS production under the catalysis of ferrite nanoparticles in the existing publications can be roughly summarized as the following (shown in Scheme 1): (1) the intrinsic Fenton reaction catalytic activity of ferrite nanomaterials, (2) external field energy enhanced Fenton reaction, and (3) the cascade reactions to generate sufficient ROS.

### Intrinsic Fenton Reaction of Ferrite

The intrinsic Fenton reaction catalytic activity is the most important mechanism of ferrite nanoparticles for ROS-mediated tumor therapy. Ferrite nanoparticles can specifically accumulate at the tumor site *via* enhanced permeability and retention effect and magnetic targeting and simultaneously release ferrous and ferric ions in tumor acidic environment to participate in the Fenton reaction with H_2_O_2_ and generate •*OH* (Wang et al., 2018). The Fenton and Fenton-like reactions can be shown as the following equations: $Fe^{2+}+H_{2}O_{2}\to Fe^{3+}+HO•+OH^{-}$ (1); $Fe^{3+}+H_{2}O_{2}\to Fe^{2+}+HO_{2}+H^{+}$ (2) (Bokare and Choi, 2014). The intrinsic catalytic activity of ferrite nanoparticles can be flexibly designed and controlled by adjusting the particles composition, size, morphology, etc.

**Figure 1:**
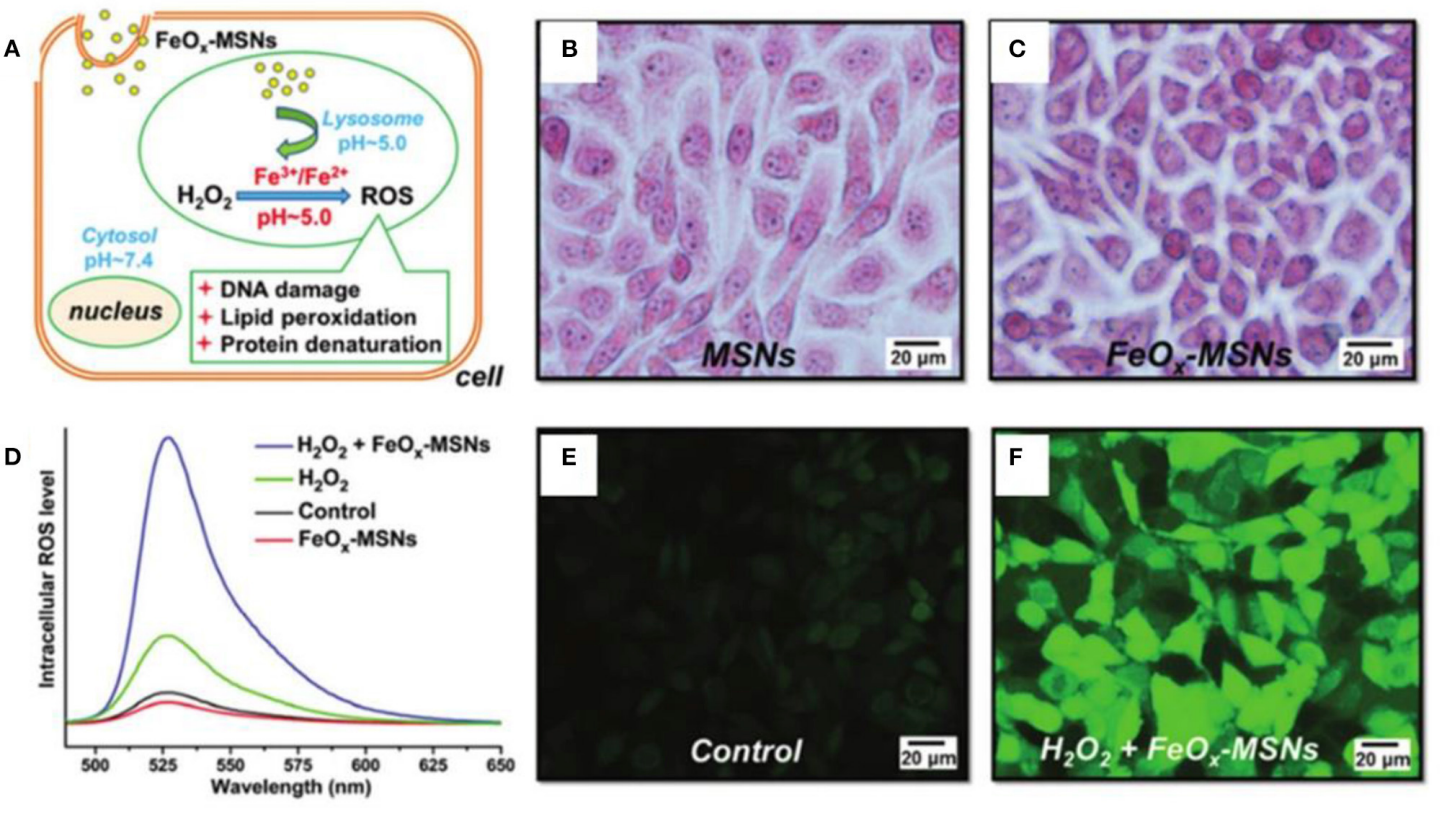
**(A)** Illustration of interactions of FeO_x_-MSNs and H_2_O_2_. Turnbull's blue stain of ZR75-30 cells after incubation with **(B)** MSNs and **(C)** FeO_x_-MSNs. **(D)** Fluorescence emission spectra of DCF in ZR75-30 cells after incubation with FeO_x_-MSNs and/or H_2_O_2_. Fluorescence images of ZR75-30 cells in the **(E)** control and **(F)** FeO_x_-MSN plus H_2_O_2_ groups. Reproduced, with permission, from Fu et al. (2015). Copyright 2015, Royal Society of Chemistry.

Wang et al. pioneered the study of magnetic nanoparticles for tumor treatment (Zhang et al., 2013). They synthesized 6 and 13 nm magnetite nanoparticles (MNPs) through a one-pot method, which possessed enzyme-mimicking activity to produce ROS efficiently for cancer theranostics. The smaller size MNPs had higher enzyme-mimicking activity, and an ~99% tumor inhibition ratio was obtained by combining with intratumoral injection of exogenous hydrogen peroxide after treatment for 17 days. The size dependence of the catalytic activity of ferrite nanoparticles was further studied by Liu and colleagues. They investigated the cytotoxic effects of small Fe_3_O_4_ nanoparticles with different diameters (6, 9, and 14 nm) on human hepatoma cell lines, SK-Hep-1 and Hep3B (Xie et al., 2016). The 9 nm Fe_3_O_4_ nanoparticles mediated mitochondria-dependent intracellular ROS generation to induce cellular mitochondrial dysfunction and necrosis, while the 14 nm Fe_3_O_4_ nanoparticles led to plasma membrane damage. Luo et al. obtained similar results that a suitable size (15.1 nm) of superparamagnetic iron oxide nanoparticles (SPIONs) enhanced the uptake amount into MCF7 cells, leading to the formation of more ROS (Zhang et al., 2020c). Promoted ROS was produced in mitochondria to destroy mitochondria by small size (7.3 nm) SPIONs, while more ROS was yield in plasma to destroy cytomembrane by larger size (15.1, 30.0 nm) SPIONs. As can be seen from the above description, the size may affect the distribution of the nanoparticles. It would be more efficient if the ferrite nanoparticles can be delivered to the desired area. Zhu et al. developed a pH-responsive iron oxides-loaded mesoporous silica nanosystem (FeO_x_-MSNs), which could deliver FeO_x_ to lysosomes and release Fe^2+^/Fe^3+^ in acidic environment to catalyze the decomposition of H_2_O_2_ to generate considerable ROS to damage breast carcinoma cells efficiently (Figure 1) (Fu et al., 2015).

**Figure 2:**
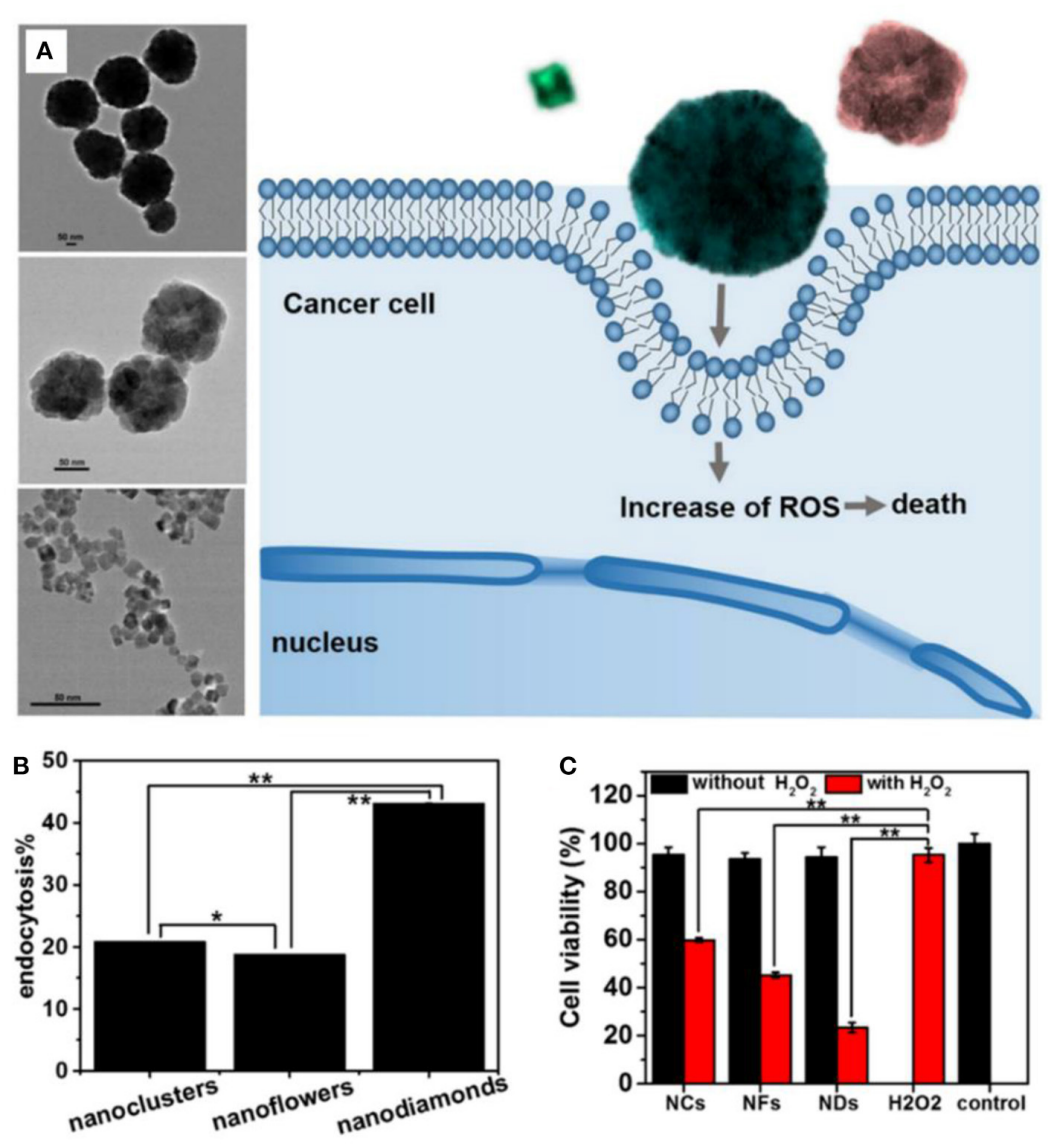
**(A)** Schematic illustration for the structural effect of Fe_3_O_4_ nanoparticles on ROS generation for cancer cell killing. **(B)** Endocytosis percentage of three kinds of Fe_3_O_4_ nanoparticles. **(C)** Cell viability incubated with Fe_3_O_4_ (1 mg/ml) alone or Fe_3_O_4_ (25 μg/ml) plus H_2_O_2_ (0.625 μM). Statistical significance, *p < 0.05, **p < 0.01. Reproduced, with permission, from Fu et al. (2017). Copyright 2017, Elsevier.

The morphology has a significant impact on the properties of nanomaterials. Li et al. fabricated Fe_3_O_4_ nanoparticles with nanocluster, nanoflower, and nanodiamond structures by tuning the pH of the hydrothermal reaction (Figure 2) (Fu et al., 2017). The structure has a great influence on peroxidase-like activity, following the order of nanocluster > nanoflower > nanodiamond. However, nanodiamonds had the highest cellular endocytosis (43.2, 20.8, and 18.8% of the added nanoparticles for nanodiamonds, nanoclusters, and nanoflowers, respectively). The cell viability data indicated that the cancer cell killing activity of the Fe_3_O_4_ nanoparticles was induced by the generated intracellular ROS through the Fenton reaction with H_2_O_2_, which was codetermined by the cell endocytosis of the nanoparticles and their enzyme-like activity.

**Figure 3:**
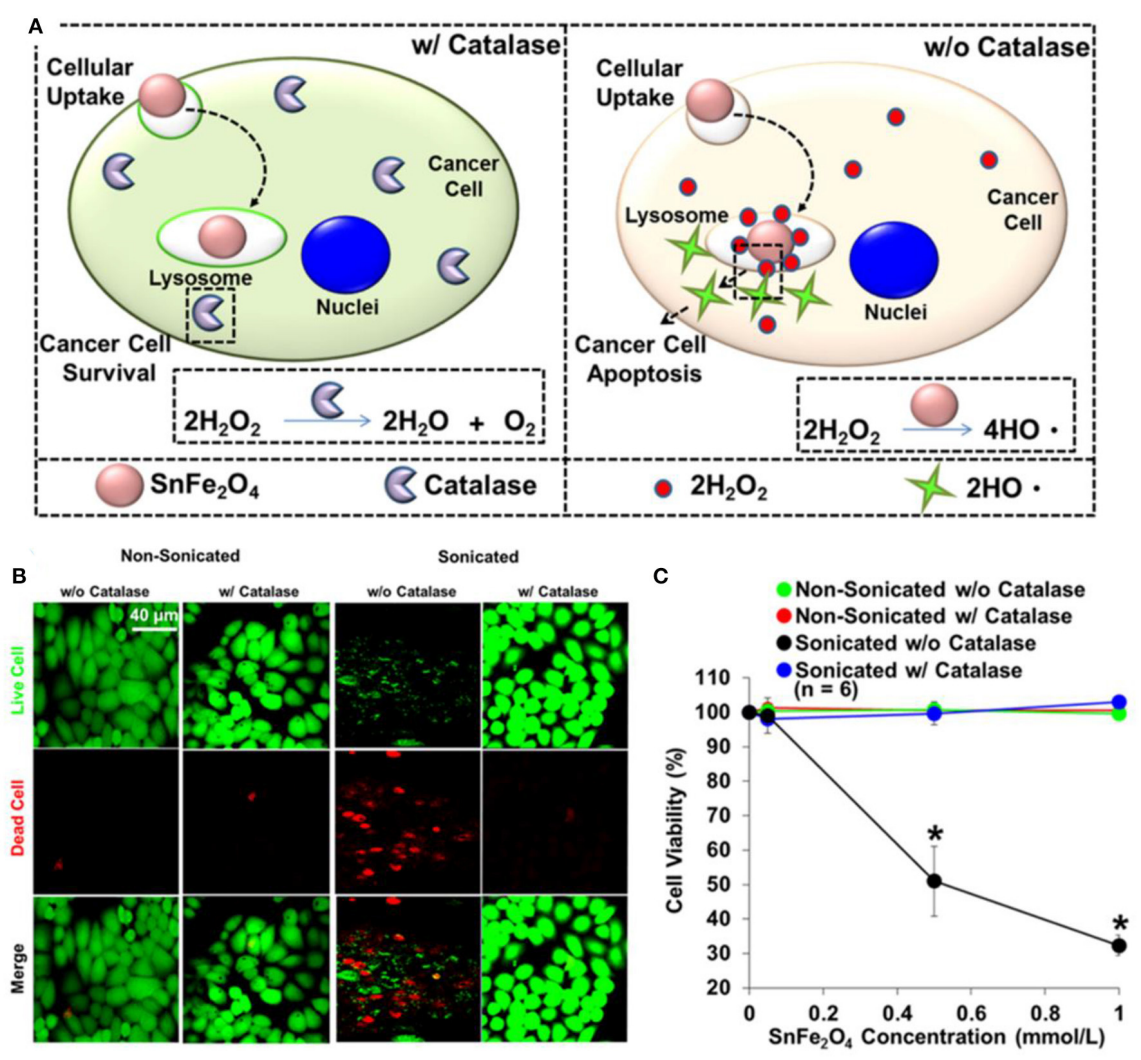
**(A)** Illustration for cytotoxic effect of SnFe_2_O_4_ nanocrystals on cancer cells. **(B)** Fluorescent images of test cells. **(C)** Corresponding quantitative results obtained using MTT. *Statistical significance indicated by *P* < 0.05. Reproduced, with permission, from Lee et al. (2017). Copyright 2016, American Chemical Society.

Non-ferrous metal species such as copper, zinc, and iridium are widely used to regulate the performance of ferrite nanoparticles. Alshamsan et al. prepared copper ferrite nanoparticles, which could induce evident oxidative stress by ROS generation and glutathione depletion, triggering the death of human breast cancer MCF-7 cells (Ahamed et al., 2016). Liao et al. also incorporated copper into ferrite nanoparticles to regulate the H_2_O_2_ catalytic ability (Kuo et al., 2020). They changed the loading amount of iron precursor concentration to control the Fe/Cu ratio of the CuFe NPs. The Combination of Fe and Cu in the oxide form could enhance the conversion of H_2_O_2_ to ROS, and the optimal Fe/Cu ratio was 2. Chuang et al. synthesized SnFe_2_O_4_ nanocrystals with sonication treatment, which could be delivered through inhalation for lung cancer therapy (Figure 3) (Lee et al., 2017). The lattice ferric ions can convert endogenous H_2_O_2_ into highly toxicity •*OH* to effectively eradicate cancer cells through heterogeneous Fenton reaction. Wang et al. treated the cancer cells with iridium and Fe^2+^ ions to biosynthesize the biocompatible iridium oxide and iron oxide nanoclusters under the redox microenvironment (Shaikh et al., 2020). Their results demonstrated that U87 and HepG2 cells incubated with Ir–Fe significantly increased the ROS generation compared to Ir ions alone, triggering the apoptosis to inhibit tumor growth. Siddiqui et al. compared the cytotoxic activity of copper oxide (CuO), iron oxide (γFe_2_O_3_), and zinc, iron, and copper oxide (CuZnFe_2_O_3_) in human breast cancer (MCF-7) cells (Siddiqui et al., 2020). The increase in ROS level could be important mechanism of metal oxide nanoparticles-induced cytotoxicity in cancer cells. The above-mentioned results fully indicated that single and multimetal oxide nanoparticles exhibited differential cytotoxic responses in cancer cells, and the ROS production could be regulated by the chemical composition of nanomaterials.

**Figure 4:**
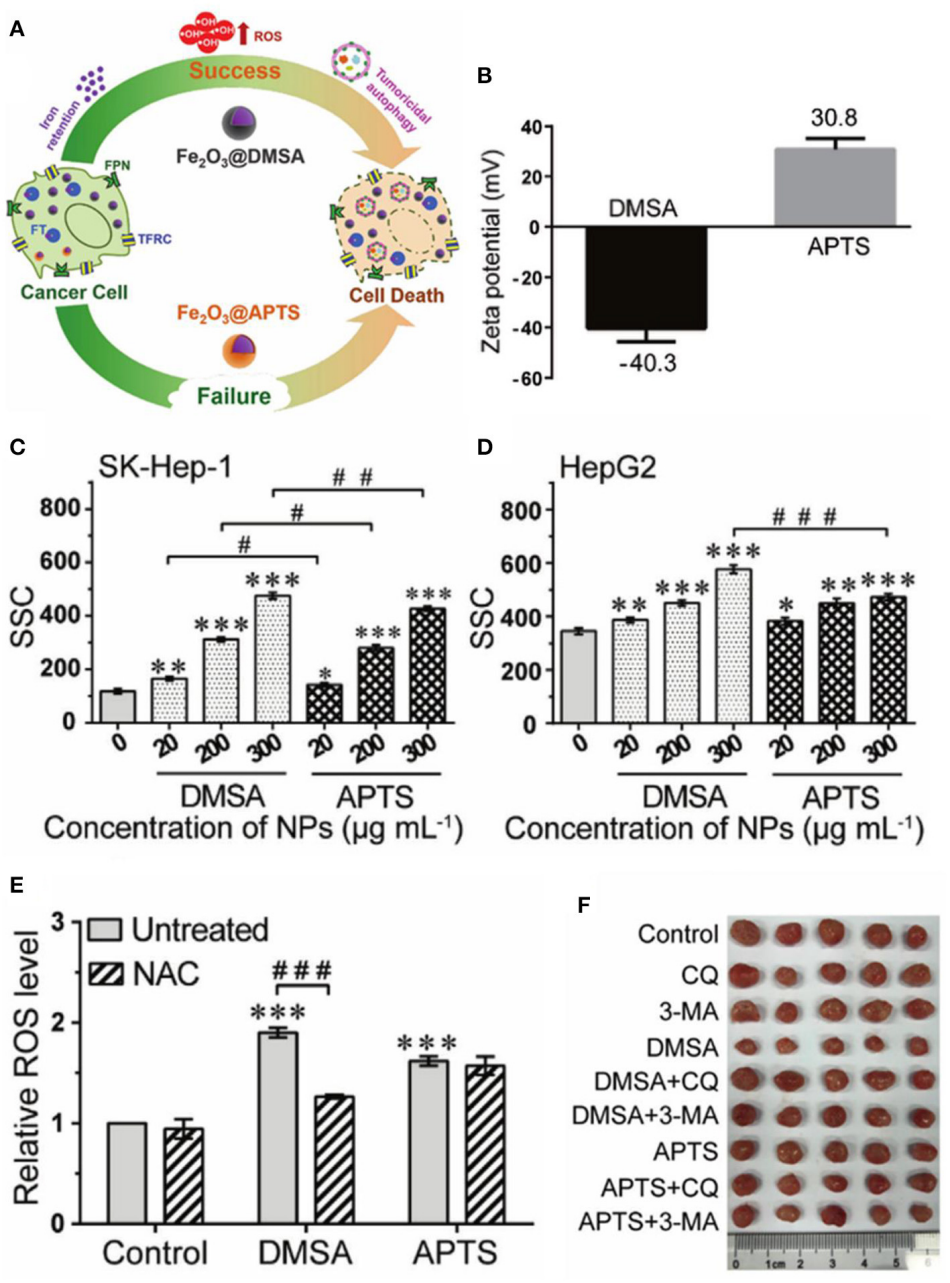
**(A)** Schematic illustration for Fe_2_O_3_@DMSA promoted ROS-induced tumoricidal autophagy. **(B)** Zeta potential of Fe_2_O_3_@DMSA and Fe_2_O_3_@APTS. **(C,D)** Cellular uptake of Fe_2_O_3_@DMSA and Fe_2_O_3_@APTS. **(E)** ROS production of SK-Hep-1 cells exposed to Fe_2_O_3_@DMSA or Fe_2_O_3_@APTS. **(F)** Photographs of tumors. Statistical significance, *p < 0.05, **p < 0.01, and ***p < 0.001 compared with control. ^#^*p* < 0.05,^*##*^*p* < 0.01, and ^*###*^*p* < 0.001 between the indicated groups. Reproduced, with permission, from Xie et al. (2020). Copyright 2020, WILEY-VCH Verlag GmbH & Co. KGaA, Weinheim.

Surface modification also plays an important role in the preparation, stability, and activity of ferrite nanoparticles. Tiku synthesized phyllanthus emblica-coated iron oxide nanoparticles (IONPAs) using a green approach (Thoidingjam and Tiku, 2019). The phyllanthus emblica could act as stabilizing agents by binding to the surfaces of the formed IONPs, so that IONPA was smaller in size with better dispersibility, leading to higher uptake in A549 lung cancer cells to produce more ROS to induce higher DNA damage and apoptosis. Small molecule coatings may significantly change the surface properties of the nanoparticles, which is needed to be considered in designing the nanoplatforms for cancer therapy. Hilt et al. observed that small molecule (citric acid, sodium phosphate, aminosilane, or dopamine) coatings could decrease surface reactivity of IONPs and inhibit ROS generation (Mai and Hilt, 2019). Conversely, Liu et al. reported that carboxy-functional Fe_2_O_3_ nanoparticles (Fe_2_O_3_@DMSA) with negative zeta potential had higher cellular uptake efficiency, which promoted intracellular iron-retention-induced ROS production but inhibited the fusion of lysosomes and autophagosomes to enhance tumoricidal autophagy for cancer therapy (Figure 4) (Xie et al., 2020). Ge et al. developed a novel ellipsoidal composite nanoplatform using a magnetic Fe_3_O_4_/Fe nanorod core enwrapped by a catalase-imprinted fibrous SiO_2_/polydopamine shell (Fe_3_O_4_/Fe@F-SiO_2_/PDA) (Chen et al., 2017a). The catalase-imprinted shell can selectively inhibit the activity of catalase to elevate H_2_O_2_ level, which could be converted into •*OH* under the catalysis of Fe ions released by Fe_3_O_4_/Fe core, triggering apoptosis to effectively kill MCF-7, 293T, and Hela tumor cells combined with the near-infrared light photothermal effect of the polydopamine layer. Targeting and responsive molecules assembled on the surface of nanoparticles can improve delivery efficiency and selectivity. Horak et al. prepared magnetic and temperature-sensitive solid lipid particles (mag. SLPs) using oleic acid-coated iron oxide, 1-tetradecanol, and poly(ethylene oxide)-block-poly(E-caprolactone), which could melt down in the tumorous tissue to produce more ROS than the non-magnetic SLPs and neat iron oxides, inducing apoptosis of Jurkat leukemic cells (Swietek et al., 2020). Sawant et al. developed novel pH responsive and mitochondria targeted poly-l-lysine-coated Fe_3_O_4_@FePt core shell nanoparticles (Mito-PANPs) (Pandey et al., 2020). Mitochondria directing triphenylphsphonium ion mediated the delivery of nanoparticles to mitochondria, enhancing ROS generation to provide multimodal therapy for glioblastomas. The gradient core–shell structure is widely used to modify the surface of functional nanomaterials. Hou et al. developed a pH-sensitive nanoreactor based on core–shell-structured iron carbide nanoparticles with amorphous Fe_3_O_4_ shells (Fe_5_C_2_@Fe_3_O_4_). The amorphous Fe_3_O_4_ shells were less stable against dissolution and able to release ferrous ions in acidic environments to generate •OH through the Fenton reaction of H_2_O_2_, effectively inhibiting the proliferation of tumor cells (Yu et al., 2019).

**Figure 5:**
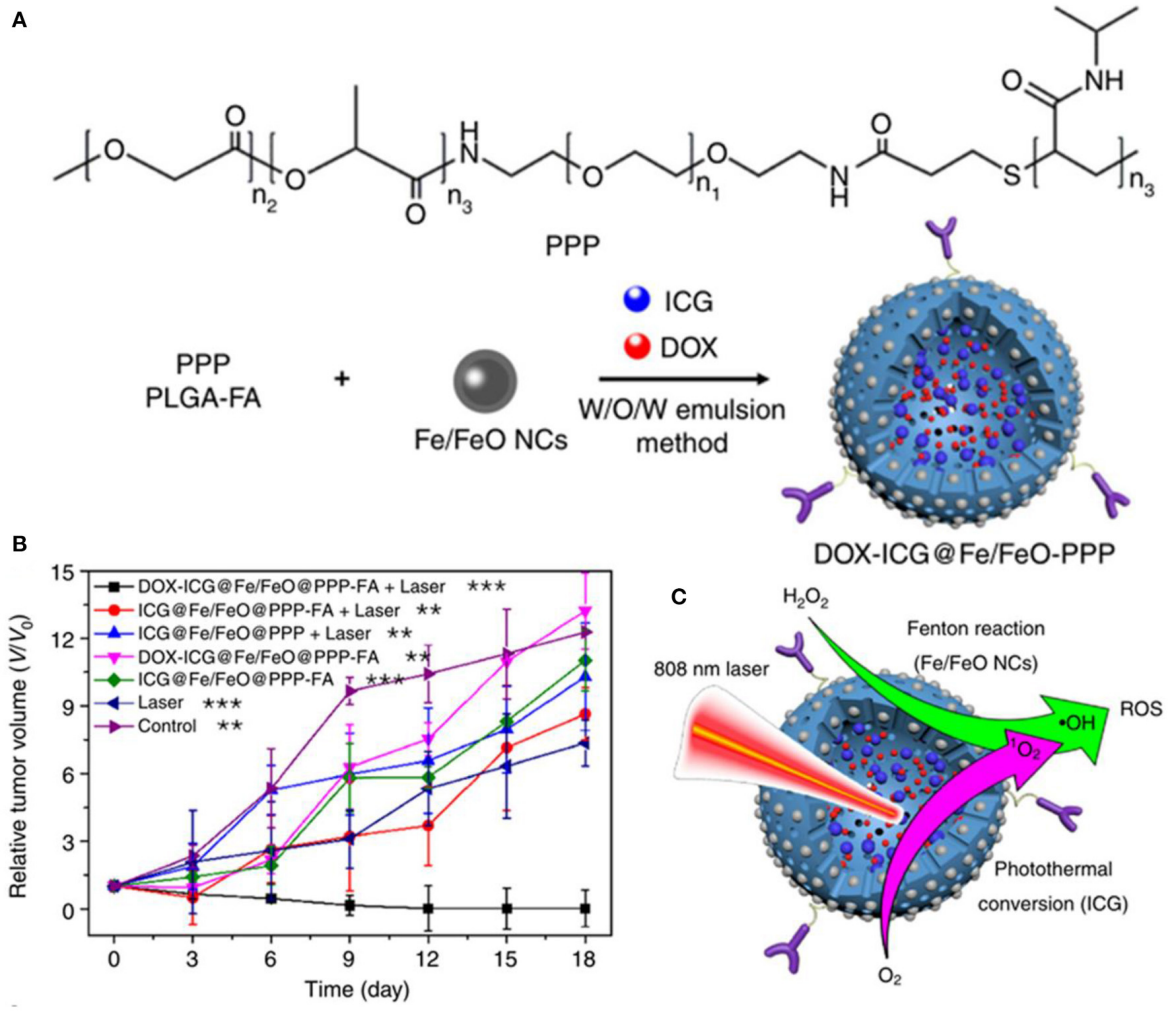
**(A)** Fabrication of DOX–ICG@Fe/FeO–PPP nanocapsules. **(B)** Volume change of tumor in different treatments. **(C)** Synergism schematic of Fenton reaction of Fe/FeO NCs with photothermal conversion (ICG). **p < 0.01, ***p < 0.001. Reproduced, with permission, from Wang et al. (2019c). Copyright 2019, Nature Publishing Group.

The effect of ROS-mediated tumor therapy can be significantly improved by combining ferrite nanoparticles with chemotherapeutic drugs, chemical or biological agents, etc. Bahadur et al. developed PEGylated mesoporous iron platinum–iron oxide composite nanoassemblies with high loading capacity of doxorubicin, which exhibited a higher efficiency of ROS generation compared to Fe_3_O_4_ and Pt under the synergistic catalytic effect of FePt and Fe_3_O_4_, resulting in efficient chemo- and thermal therapy for Hela cancer cells (Sahu et al., 2015). Yeh et al. presented an H_2_O_2_-loaded ultrasound contrast agent H_2_O_2_/Fe_3_O_4_-poly(D,L-lactide-co-glycolic acid (PLGA) polymersome, which could yield sufficient •*OH* through the Fenton reaction of encapsulated H_2_O_2_ and Fe_3_O_4_, completely removing the malignant tumors in a non-thermal process (Li et al., 2016). Watanabe et al. investigated the combined effects of Fe_3_O_4_ nanoparticles with chemotherapeutic agents (rapamycin or carboplatin) on prostate cancer cells *in vitro* (Kojima et al., 2018). Synergistic effect of Fe_3_O_4_ NPs was observed in DU145 cells with carboplatin and in PC-3 cells with rapamycin, increasing intracellular ROS levels to decrease cancer cell viability significantly. Hou et al. designed a PLGA-polymer matrix coated with Fe/FeO core–shell nanocrystals and coloaded with chemotherapy drug and photothermal agent (DOX-ICG@Fe/FeO-PPP-FA nanocapsules), which could *in situ* overproduce ROS by reacting with endogenous H_2_O_2_ in tumors, overcoming the tumor hypoxia-related resistance of chemotherapy and photodynamic therapy (Figure 5) (Wang et al., 2019c). Liu et al. developed a facile synergistic nanoplatform (NanoTRAIL) using iron oxide cluster and tumor necrosis factor-related apoptosis-inducing ligand (TRAI/Apo2L), which could release iron oxide NPs to generate ROS to provoke JNK-autophagy-dependent DR5 upregulation, leading to enhancing TRAIL/Apo2L-induced apoptosis in colorectal cancer (Shi et al., 2020). Thenmozhi prepared PVP-coated iron oxide nanoparticles loaded with *syzygium aromaticum* extract, which could induce oxidative stress *via* ROS formation, enhancing MCF-7 breast cancer apoptosis (Thenmozhi, 2020). This interesting study of the application of biomass *syzygium aromaticum* extract can provide a useful reference for the application researches of traditional Chinese medicine.

The applicability of ROS-mediated treatment to different types of cancers has been extensively verified. Ahamed et al. prepared spherical iron oxide nanoparticles with a smooth surface and an average diameter of 23 nm, which could induce the reactive oxygen species generation in HepG2 and A549 cancer cells, upregulating tumor suppressor gene p53 and caspase-3 and caspase-9 apoptotic genes to trigger cancer cells apoptosis (Ahamed et al., 2013). In subsequent research, they found that the MCF-7 cells were slightly more sensitive to nickel ferrite nanoparticles than liver HepG2 cells induced by reactive oxygen species (Ahamed et al., 2015). Gokduman synthesized magnetite iron oxide nanoparticles with a diameter of ~20 nm, which could increase intracellular ROS, enhancing the anticancer activity of cisplatin by increasing the apoptosis of the cisplatin-resistant ovarian cancer cells (OVCAR-3 and SKOV-3) (Gokduman, 2019). Rajesh et al. developed a hybrid magnetic microsphere system (Fe_3_O_4_@LEC-CUR-PLGA-MMS) using iron oxide nanoparticle (Fe_3_O_4_ NP), lecithin (LEC), curcumin (CUR), PLGA, and polyethylene glycol (PEG) (Ayyanaar et al., 2020). The Fe_3_O_4_ could catalyze the generation of ROS in an H_2_O_2_ environment to release the CUR, showing greater cytotoxicity against A549 and HeLa S3 cells. Salehzadeh et al. synthesized Fe_3_O_4_@CPTMOS/TP NPs, which had an effect on induction of apoptosis and inhibition of the growth of gastric AGS cancer cells by increasing ROS production in the treated cells (Habibzadeh et al., 2020). The IC_50_ value in AGS cells was estimated to be 95.65 μg/ml. Ramalingam et al. prepared hematite α-Fe_2_O_3_ by wet chemical method, which showed dose-dependent anticancer activity against human metastatic ovarian cancer (OC) by inducing ROS generation, damaging the mitochondrial membrane, and triggering the apoptosis of OC PA-1 cells (Ramalingam et al., 2020). Pourahmad et al. investigated the effect of SPIONs on the oral tongue squamous cell carcinoma (OTSCC) (Jahanbani et al., 2020). SPIONs were able to increase the level of ROS formation in cancerous mitochondria to selectively initiate ROS-mediated apoptosis of SCC cells. As can be seen from the above descriptions, ROS-mediated therapies based on ferrite nanoparticles have broad applicability to a wide range of cancers.

**Figure 6:**
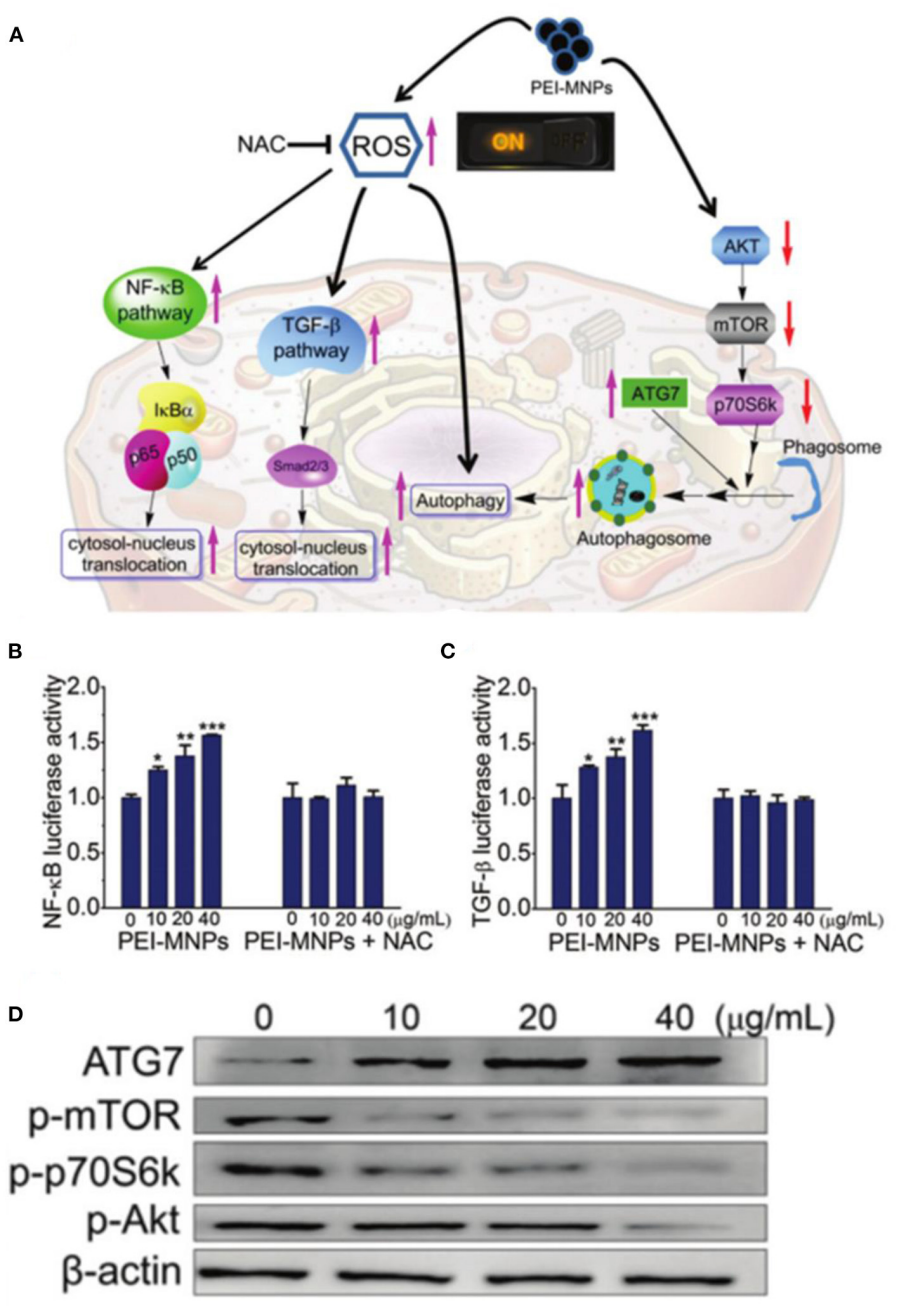
**(A)** Schematic illustration for roles of ROS on PEI-MNPs elicited responses in cancer cells. **(B,C)** PEI-MNPs induced overproduction of ROS, triggering the activation of NF-κB and TGF-β pathways. **(D)** Western blotting experiments of the cancer cells treated with PEI-MNPs. *P < 0.05;**P < 0.01;***P < 0.005 vs controls. Reproduced, with permission, from Man et al. (2020). Copyright 2020, Royal Society of Chemistry.

The study of mechanisms and pathways for ROS-mediated cancer therapy based on ferrite nanoparticles has also attracted the attention of researchers. Liu et al. investigated the molecular mechanism of SPIONs induced cancer-cell-specific cytotoxicity through DNA microarray and bioinformatics analyses (He et al., 2016a). SPIONs can interfere with the mitochondrial electron transport chain to induce the formation of ROS, triggering cytotoxicity to the cancer cells. Han et al. reported that ultrasmall 9 nm Fe_3_O_4_ NPs could effectively internalize into cells and locate in the nucleus and induce ROS production and oxidative damage by disturbing the expression of antioxidant-related genes, suggesting a potential antitumor application (Ye et al., 2020). Ma et al. demonstrated polyethyleneimine-coated Fe_3_O_4_ magnetic nanoparticles (PEI-MNPs), which could contribute to ROS overproduction by the Fenton reaction, resulting in autophagy induction *via* mTOR-Akt-p70S6 K and ATG7 signaling pathways to kill cancer cells (Figure 6) (Man et al., 2020). Further research on the mechanism will be beneficial to the development of more effective ferrite nanoparticle-based therapeutic agents.

### External Field Enhanced Fenton Reaction

Only relying on the intrinsic Fenton reaction catalytic activity of ferrite nanomaterials often requires a high concentration to generate enough ROS to kill tumor cells, which may increase the burden of iron removal based on the kidney and liver and cause adverse damage to the body (Ranji-Burachaloo et al., 2018). External electromagnetic waves such as X-ray, near-infrared light and alternating magnetic field can be absorbed by the ferrite-based nanoplatform to improve the production of reactive oxygen species (Laurent et al., 2011; Pilar Vinardell and Mitjans, 2015; Xiong et al., 2019). Ultrasound, a typical high-frequency mechanical wave, can also be used as an external energy source. Gorgizadeh et al. synthesized a nickel ferrite/carbon nanocomposite (NiFe_2_O_4_/C) as sonosensitizer (Gorgizadeh et al., 2019). Radiation of ultrasound into NiFe_2_O_4_/C effectively induced cavitation formation and ROS production, resulting in remarkable efficacious recovery in mouse melanoma cancer model by intratumorally injection at dosage of ~100 mg kg^−1^.

**Figure 7:**
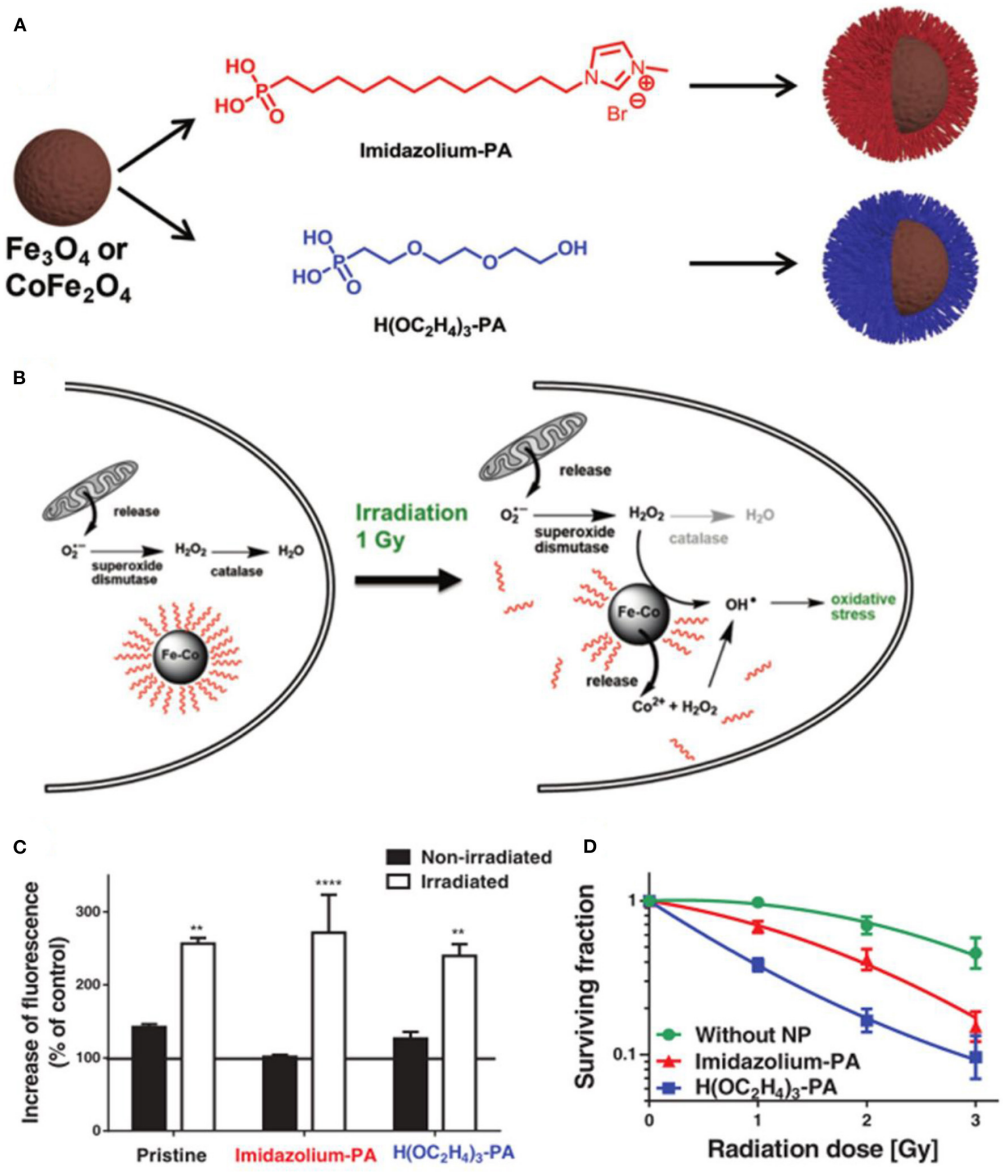
**(A)** Fabrication of PA-SAM functionalized Fe_3_O_4_ and CoFe_2_O_4_ MNPs. **(B)** Mechanisms of ROS generation under X-ray irradiation. **(C)** Determination of ROS concentration in MCF-7 cells. **(D)** Survival curves of MCF-7 cells incubated with functionalized CoFe_2_O_4_ MNPs. **P < 0.01, ****P < 0.001. Reproduced, with permission, from Klein et al. (2018). Copyright 2018, WILEY-VCH Verlag GmbH & Co. KGaA, Weinheim.

Kryschi et al. first studied the citrate-coated superparamagnetic iron oxide nanoparticles as X-ray radiosensitizer (Klein et al., 2012). The increased catalytically active iron oxide nanoparticle surfaces can enhance the ROS generation for about 240% under the X-ray exposure. In subsequent research, they synthesized 9–20 nm (γ*F*_*e*_2_*O*_3_)1−*x*_(*F*_*e*_3_*O*_4_)*x*_ surface stabilized with citrate or malate anions, which can drastically enhance the ROS concentration of more than 300% *via* the Fenton reaction in 1 Gy X-ray-irradiated tumor cells (Klein et al., 2014). Hadjipanayis et al. showed that cetuximab-conjugated iron oxide nanoparticles (cetuximab-IONPs) could sensitize ionizing radiation therapy by increasing ROS formation and DNA double strands breaks (Bouras et al., 2015). Hilt et al. developed a cell-penetrating peptide functionalized iron oxide nanoparticle (TAT-Fe_3_O_4_) to increase the efficacy of radiation therapy (Hauser et al., 2016b). Radiation promoted the production of the superoxide anion in mitochondria, which was further converted to hydrogen peroxide by superoxide dismutase, and the generated H_2_O_2_ could be catalyzed to the highly reactive hydroxyl radical by the Fenton reaction with iron oxide nanoparticles for the enhancement of radiation therapy. Kryschi et al. synthesized functionalized superparamagnetic magnetite (Fe_3_O_4_) and Co-ferrite (CoFe_2_O_4_) nanoparticles with self-assembled monolayer coatings, which have long-term stability and could be activated through X-ray exposure with a single dosage of 1 Gy to induce ablation of the surface coverage and release either Fe^2+^ or Co^2+^ ions, enhancing the production of the highly hydroxyl radical *via* the Fenton reaction to kill the cancerous MCF-7 cells efficiently (Figure 7) (Klein et al., 2018).

**Figure 8:**
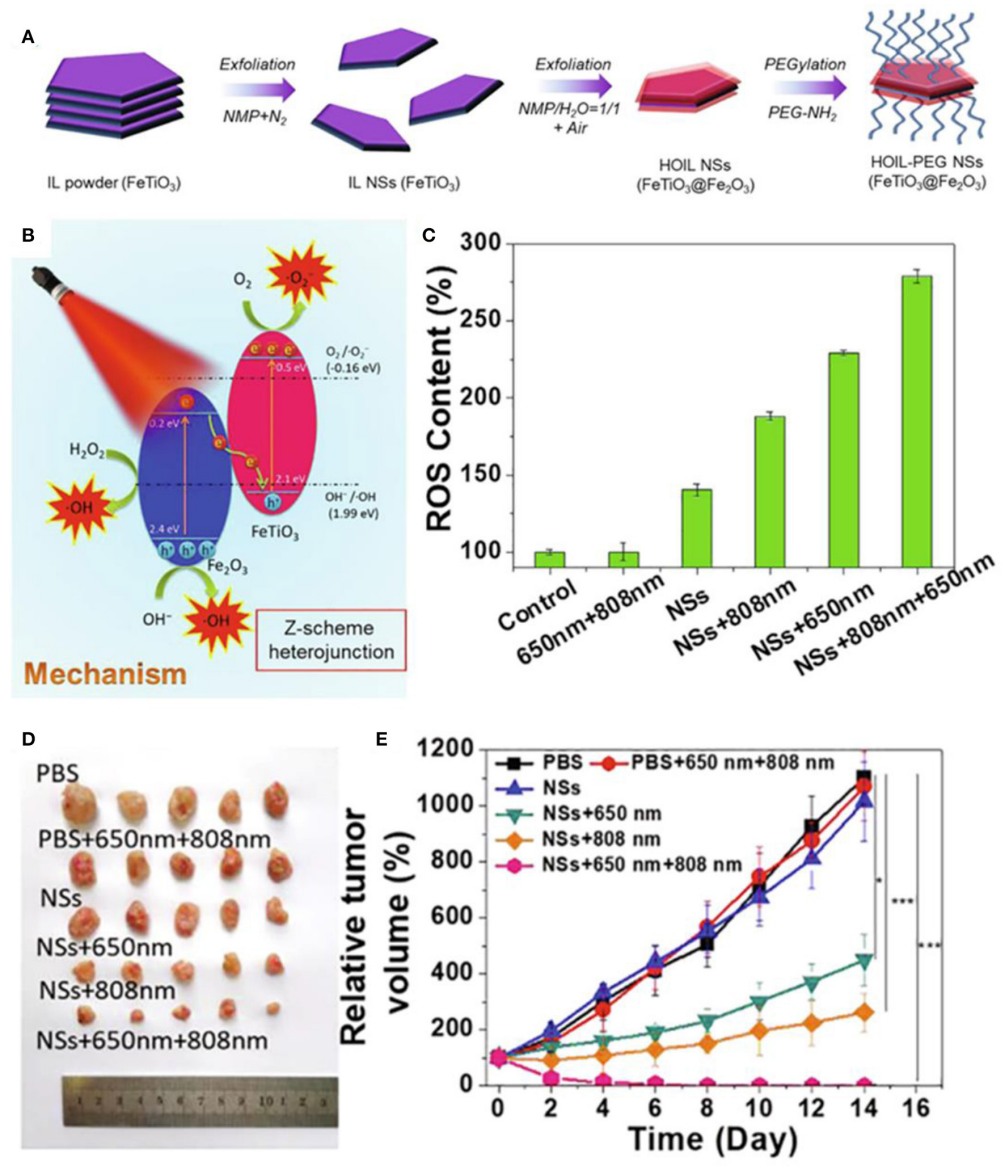
**(A,B)** Schematic illustration of fabrication and therapy mechanism of HOIL-PEG NSs. **(C)** ROS content of A549 cells after treated. **(D)** Morphology of representative tumors. **(E)** Tumor volume of A549 tumor-bearing nude mice after treatment. Statistical values are indicated in figures according to the following scale: *P < 0.05, **P < 0.01 and ***P < 0.001. Reproduced, with permission, from Ou et al. (2020). Copyright 2020, Elsevier.

Light waves are also widely used as external field energy sources. Near-infrared light irradiation can be efficiently converted into heat to enhance ROS generation. Miao et al. synthesized Zn^2+^-doped magnetic nanoparticles *via* hydrothermal route, which revealed excellent photothermal effect to generate localized heat and increase the dissolution of magnetic nanoparticles in the acid medium to enhance ROS generation upon a near-infrared (NIR) light irradiation, inducing cancer treatment (Qi et al., 2016). Chen et al. confirmed that the bacterial magnetic nanoparticles could induce increased level of intracellular ROS along with heat under near-infrared light irradiation to trigger an efficient tumor cell kill (Chen et al., 2016). Dong et al. fabricated a nanoplatform based on iron oxide nanoparticles, indocyanine green, and hyaluronic acid (IONPs-ICG-HA) (Wang et al., 2019a). The iron oxide could convert intracellular H_2_O_2_ to generate fatal reactive oxygen species through Fenton reaction, which could be boosted by increased temperature of photothermal effect of ICG, enhancing synergistic phototherapy in cancer treatment. Li et al. developed a tumor-targeting iron sponge γGDYO-Fe_3_O_4_-CREKA (TTIS) nanocomposite, which could accelerate the release of iron ions to enhance the efficiency of the Fenton reaction and generate more ROS by the heat produced in the process of photothermal therapy (Min et al., 2020). You et al. designed a more stable and high-ROS-yielding Pt/Fe_3_O_4_@SP-PLGA lipo-polymersom, which could significantly increase the generation of •*OH* for ROS-mediated cancer therapy through the reaction between succinic peroxide (SP) and iron oxide under NIR irradiation (You et al., 2019). Photosensitizers can be activated by laser irradiation to improve the electron–hole pairs separation efficacy and redox potentials, leading to strong ability in generating ROS. Ji et al. prepared 2D ultrathin Z-scheme highly oxidized ilmenite nanosheets (FeTiO_3_@Fe_2_O_3_) with much strong oxidation and reduction potentials in the valence band (VB) of Fe_2_O_3_ and the conduction band (CB) of FeTiO_3_, which could enhance the generation of $O_{2}^{-}$ from O_2_ on the CB of FeTiO_3_ and •*OH* from H_2_O_2_ on the VB of Fe_2_O_3_ for antitumor therapy under irradiation of 650 nm laser (Figure 8) (Ou et al., 2020).

**Figure 9:**
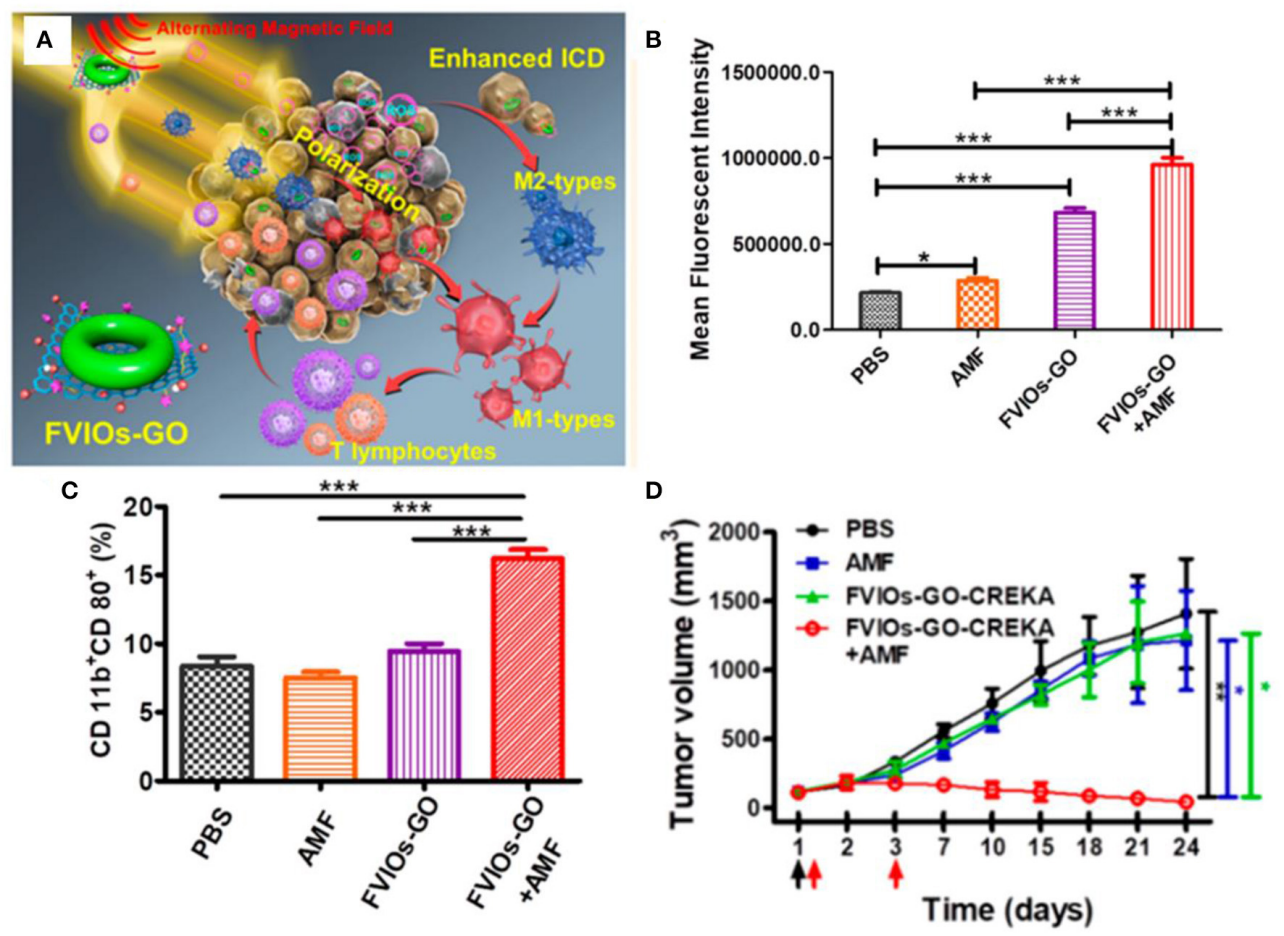
**(A)** Schematic illustration for FVIOs-GO-mediated MTD by combination of a heating effect and ROS-related immunologic effect. **(B)** Quantification of ROS generation of 4T1 breast cancer cells. **(C)** Quantification of M1 macrophages for treatments. **(D)** Tumor volume vs. days after treatments. *0.01 < *P* < 0.05;**0.001 < *P* < 0.01;***P < 0.001. Reproduced, with permission, from Liu et al. (2020). Copyright 2020, American Chemical Society.

Ferrite nanoparticles have unique magnetic heating transfer efficiency to generate heat, enhancing the effect of ROS-mediated cancer therapy (Johannsen et al., 2010; Silva et al., 2011). The alternating magnetic field is the most commonly used due to its large penetration. Hilt et al. showed that peptide-conjugated magnetic nanoparticles (TAT-IONP) could increase cellular ROS generation in both A549 and H358 cell lines upon exposure to an alternating magnetic field, resulting in an increase in apoptosis *via* the Caspase 3/7 pathways (Hauser et al., 2016a). Orel et al. designed magnetic nanodots composed of doxorubicin-loaded Fe_3_O_4_ nanoparticles, which could release more free iron to promote the formation of highly reactive oxygen species combined with electromagnetic fields, achieving remote modulation of redox state of Walker-256 carcinosarcoma tumor for cancer nanotherapy (Orel et al., 2018). Lin et al. synthesized magnetic hydroxyapatite nanoparticles by coprecipitation with the addition of Fe^2+^ (mHAP), which could increase intracellular ROS concentration to cause DNA damage of HepG2 cells with possible MKK3/MKK6 and ATF-2 of p38 MAPK inhibition under exposure to alternating magnetic field (Yang et al., 2018). Zhang et al. designed a magnetic hydrogel nanozyme utilizing PEGylated Fe_3_O_4_ nanoparticles and a-cyclodextrin, which could enhance tumor oxidative stress level by generating more ROS through promoted peroxidase-like enzymatic activity of Fe_3_O_4_ nanozyme at 42 hyperthermia induced by a non-invasive external alternating current magnetic field (Wu et al., 2019). Fan et al. demonstrated a biocompatible elaborate ferrimagnetic vortex-domain iron oxide nanoring and graphene oxide hybrid nanoparticle (FVIOs-GO-CREKA), which had high thermal conversion efficiency to significantly amplify the generation of ROS under an alternating magnetic field, promoting macrophage polarization to proinflammatory M1 phenotypes and elevating tumor-infiltrating T lymphocytes to provoke a strong immune response at a physiological tolerable temperature below 40 in a hypoxic tumor microenvironment (Figure 9) (Liu et al., 2020). Hilger et al. reported that the magnetic heating treatment could induce more production of ROS and alter messenger RNA (mRNA) expression of Ki-67, TOP2A, and TPX2, resulting in reducing tumor volumes superior to that of extrinsic heating (hot air) significantly (Ludwig et al., 2017). The effect of treatment under static magnetic field has also attracted the interest of researchers. Pazik et al. investigated the viability of canine mastocytoma tumor cells cultured with cobalt–manganese ferrite nanoparticles (Co_0.2_Mn_0.8_Fe_2_O_4_) under 0.5 T static magnetic field (Marycz et al., 2017). The nanoparticles and magnetic field increase the temperature of tumor cells and the formation of reactive oxygen species, inducing apoptotic response.

**Figure 10:**
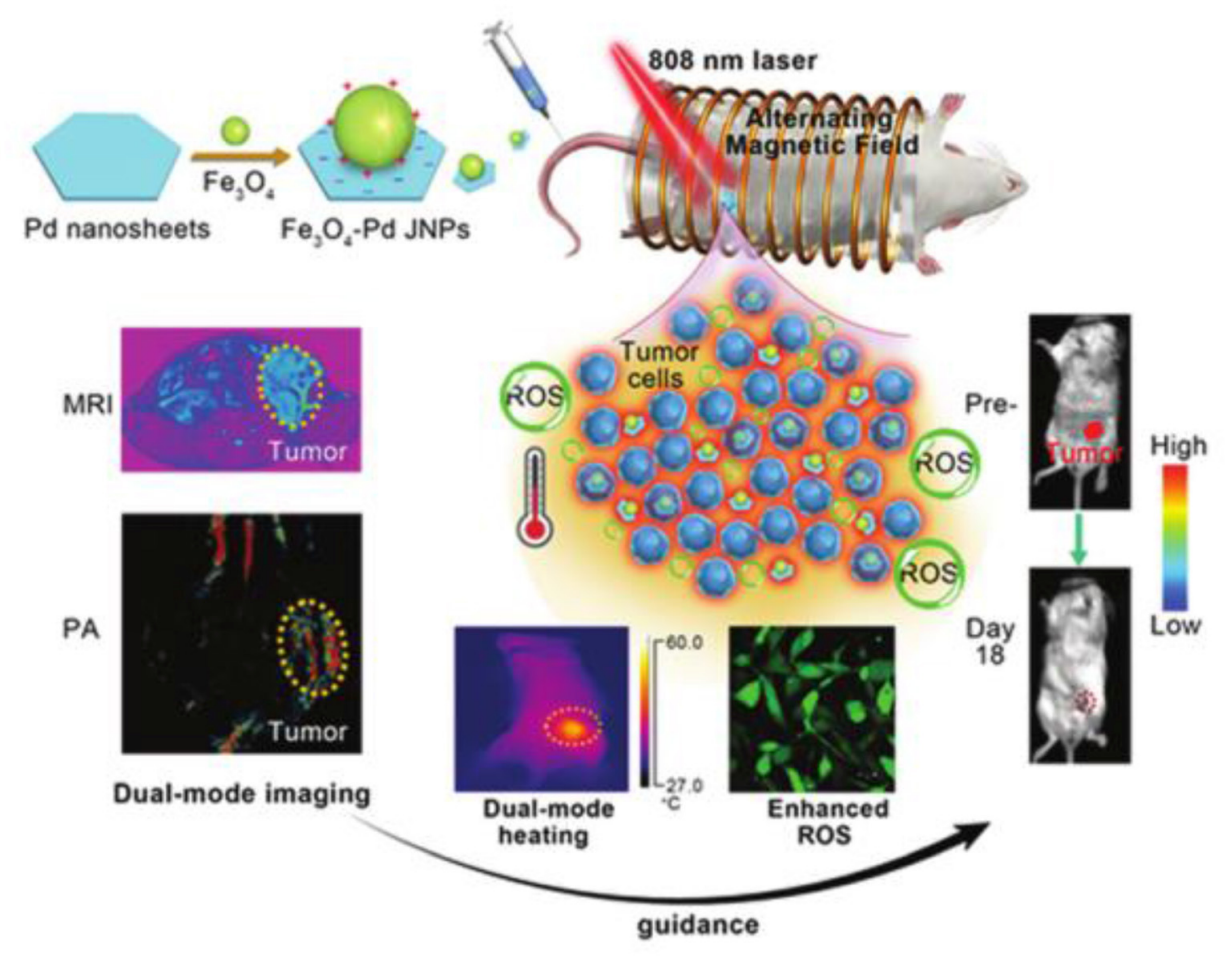
A schematic illustration for Fe_3_O_4_-Pd JNPs enhanced ROS-mediated antineoplastic therapy. Reproduced, with permission, from Ma et al. (2019). Copyright 2019, Royal Society of Chemistry.

Multifield coupling can often produce better synergistic therapeutic effects. Hassan et al. designed nanohybrid using nanoflower-like iron oxide and spiky copper sulfide shell (IONF@CuS), which could efficiently convert light and magnetic stimulation into heat and form concurrent reactive oxygen species upon laser irradiation for a tri-therapeutic strategy merging magnetic hyperthermia and photothermal and photodynamic therapy (Curcio et al., 2019). Fan et al. designed biocompatible Fe_3_O_4_-Pd Janus nanoparticles, which could enhance ROS generation due to the interface synergistic effect in producing hydroxyl radicals by Fe_3_O_4_ nanoparticle-based Fenton reaction and Pd nanosheet-based catalytic properties under external alternating magnetic field plus laser irradiation, exhibiting a high tumor-inhibition efficacy [100% tumor inhibition rate at a dose of 6 mg kg^−1^ under alternating magnetic field (AMF) (300 kHz; 300 Oe) and laser (808 nm; 0.5 W cm^−2^)] toward 4T1 orthotopic breast tumor (Figure 10) (Ma et al., 2019). Sharma et al. developed manganese doped-iron oxide nanoclusters, which could trigger heat-induced enhancement of the Fenton reaction for the generation of •*OH* under the dual application of magnetic hyperthermia and photothermal stimulation, resulting in a remarkable anticancer effect mediated by ROS-dependent apoptosis *via* the mitochondrial pathway (Gupta and Sharma, 2020).

### Cascade Reactions Increased ROS

The rapid growth of the tumor tissues and the incomplete blood vessels lead to a hypoxia environment within solid tumors (Knowles and Harris, 2001). The concentration of intratumoral H_2_O_2_ is generally considered to be as low as 50–100 μ*M*, which is not high enough to generate an effective amount of hydroxyl radicals for a satisfactory cancer therapy (Chen et al., 2017b). Intratumoral injection of hydrogen peroxide is an effective method to increase the ROS-mediated tumor therapeutic effect (Zhang et al., 2013). However, this method has poor controllability and safety, causing damage to the surrounding healthy tissues. The cascade reactions have shown a good prospect in overcoming the tumor hypoxia and increasing the ROS production.

**Figure 11:**
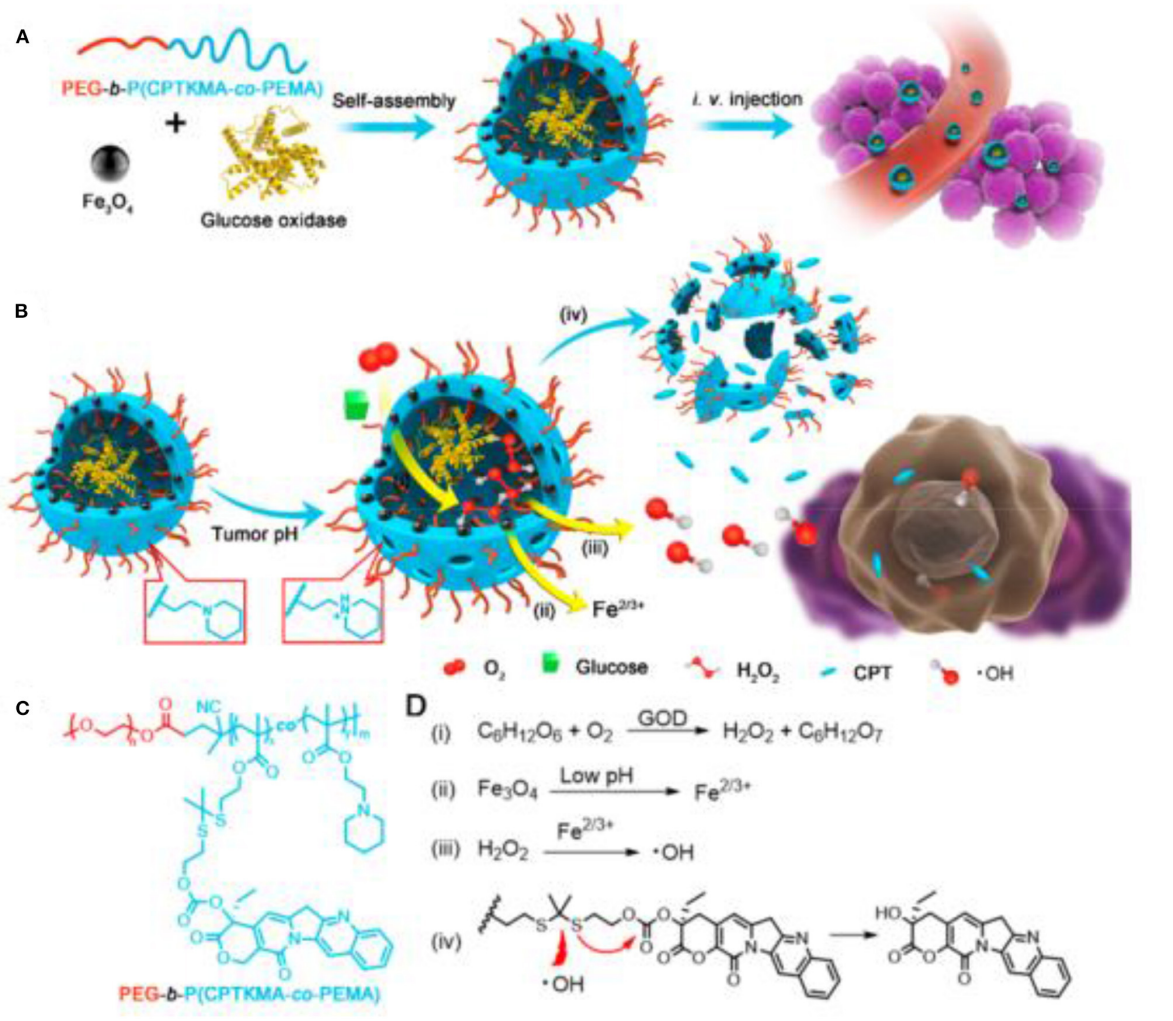
Schematic illustration for **(A)** preparation of polymersome nanoreactors and **(B)** cascade reactions in the nanoreactors. **(C)** Chemical structure of PEG-b-P(CPTKMA-co-PEMA). **(D)** Cascade reactions equations occurring in the nanoreactors. Reproduced, with permission, from Ke et al. (2019). Copyright 2019, American Chemical Society.

The most commonly used strategy is to generate more intratumoral hydrogen peroxide *in situ* through cascade reactions for the subsequent Fenton reaction. The β*-lapachone* was used earlier in such cascade reactions, which could undergo redox cycles to generate high H_2_O_2_ levels inside cancer cells. Gao et al. developed pH-responsive superparamagnetic iron oxide nanoparticles (SPION micelles), which could selectively release iron ions in tumor acidic environment to react with H_2_O_2_ generated from β*-lapachone* to produce 10-fold highly active hydroxyl radicals, displaying a synergistic efficacy for cancer treatment with ROS-generating anticancer drug (Huang et al., 2013). In another similar study, Chen et al. constructed a nanomedicine by encapsulating β*-lapachone* (La) and IONPs into the hydrophobic core of nanostructure formed by polyprodrug and polymer, which could be internalized by tumor cells and disintegrated in acidic environment to release La and iron ions (Wang et al., 2019b). The released La generated massive H_2_O_2_ through the catalysis of the nicotinamide adenine dinucleotide (phosphate) [NAD(P)H]: quinone oxidoreductase 1 (NQO1), which would further be converted to highly toxic •*OH* by Fenton reaction with iron ions, resulting in improved antitumor activity. Ascorbic acid, a known antioxidant, can also be used to produce endogenous H_2_O_2_. Wang et al. synthesized Fe_3_O_4_@C nanoparticles modified with folic acid (Fe_3_O_4_@C-FA), which could create hydroxyl radicals from H_2_O_2_ yielded by the exogenous ascorbic acid, inducing the selective killing of cancer cells owing to ROS accumulation in human prostate cancer PC-3 cells (An et al., 2013). In a similar study, as low as 0.1 mM exogenous vitamin C was catalyzed by iron oxide nanoparticle to generate H_2_O_2_ followed by ROS production in the form of hydroxyl/superoxide radicals, inducing effective tumor cell death (Pal and Jana, 2020). Cisplatin is also commonly used as cascade reaction trigger agent. Lin et al. constructed self-sacrificing iron oxide nanoparticles with cisplatin (IV) prodrug (FePt-NP2), which could release cisplatin and Fe^2+^/Fe^3+^ (Ma et al., 2017). The released cisplatin could activate nicotinamide adenine dinucleotide phosphate (NADPH) oxidase to trigger oxygen to generate superoxide radical, which could be further dismutated by superoxide dismutase to form downstream H_2_O_2_. The generated H_2_O_2_ would be catalyzed by Fe^2+^/Fe^3+^ to the toxic hydroxyl radicals, causing ROS-mediated oxidative damages to lipids, proteins, and DNA and inducing tumor cell apoptosis. This strategy was also adopted by Chen et al. to design cisplatin-loaded Fe_3_O_4_/Gd_2_O_3_ hybrid nanoparticles with conjugation of lactoferrin and RGD dimer (FeGd-HN@Pt@LF/RGD2), which could release cisplatin, Fe^2+^, and Fe^3+^ after endocytosis in the endosomes, leading to high inhibition efficacy on orthotopic brain tumors (Shen et al., 2018). Ni et al. synthesized FA/Pt+si-GPX4@IONPs for gene treatment of glioblastoma (Zhang et al., 2020b). The cascade reactions triggered by Pt laid the foundation for efficient ROS production to induce a combination of ferroptosis and apoptosis. Another cascade reaction strategy developed by Shi et al. reported a sequential catalytic nanomedicine using natural glucose oxidase and synthetic ultrasmall Fe_3_O_4_ nanoparticles to integrate into the large mesopores of dendritic mesoporous silica nanoparticles (GFD NCs) (Huo et al., 2017). The glucose oxidase (GOD) released from nanocatalysts could deplete the glucose to produce considerable amounts of H_2_O_2_, which could be converted into highly toxic hydroxyl radical through Fenton-like reaction catalyzed by Fe_3_O_4_ nanoparticles to trigger the apoptosis and death of tumor cells. This strategy was further studied by Ge et al. They engineered ultrasmall iron oxide nanoparticles (USIONs) and GOD-coloaded PEG-b-P(CPTKMA-co-PEMA) polymersomes nanoreactors (Fe/G@R-NRs), which could occur cascade reactions including glucose consumption to generate H_2_O_2_ by GOD, production of •*OH* through Fenton reaction between H_2_O_2_ and iron ion released by USIONs, and ·OH-triggered rapid release of polyprodrug for orchestrated cooperative cancer therapy including starving therapy, chemodynamic therapy, and chemotherapy (Figure 11) (Ke et al., 2019). Xu et al. developed glucose oxidase and polydopamine-functionalized iron oxide nanoparticles (Fe_3_O_4_@PDA/GOx NPs), in which the enzymatic activity of GOx was stably retained due to the excellent biocompatibility of polydopamine (Zhang et al., 2019). For cancer cells incubated with the 200 nm NPs, the •*OH* accumulation within the cells was about 2-fold higher than that with 20 nm NPs treatment, efficiently inducing the apoptosis of cancer cells. Peroxide can be employed as potent H_2_O_2_ supplier to sustain the ferrite nanoparticles-mediated Fenton reaction. Shi et al. constructed 2D multifunctional therapeutic nanoreactors by conjugating iron oxide nanoparticles and calcium peroxide onto niobium carbide (Nb_2_C-IO-CaO_2_) (Gao et al., 2019). The CaO_2_ could react with H_2_O to produce H_2_O_2_ in the acidic tumor microenvironment, which was subsequently disproportionated into highly toxic •*OH* by the IO nanoparticles for inducing tumor cell death. With laser irradiation, graphene oxide can produce more reactive graphene radicals to enhance the ROS formation. Huang et al. developed a near-infrared absorbing nanoagent using graphene oxide loaded with iron hydroxide/oxide (GO-FeO_x_H) *via* one-step electrooxidation (He et al., 2017). The electron transfer from GO to the Fe^3+^ of FeO_x_H could promote the reaction with O_2_ to generate superoxide anion radicals under NIR light irradiation, which would be converted into

H_2_O_2_ through disproportionation reaction. The generated H_2_O_2_ then underwent a reaction with Fe^2+^ of FeO_x_H to produce amplified hydroxyl radicals, triggering near-infrared activated ROS-mediated photodynamic therapy.

**Figure 12:**
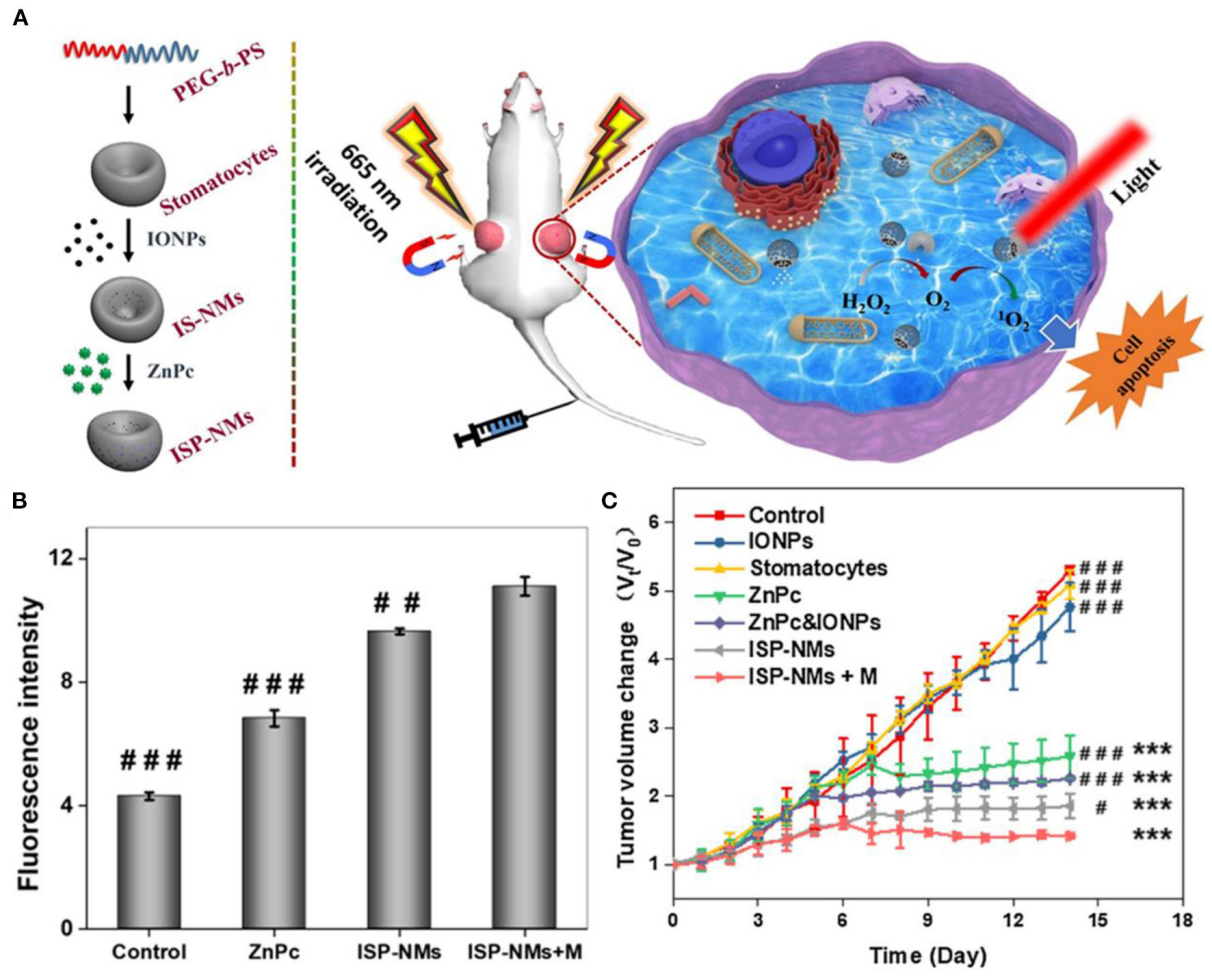
**(A)** Schematic diagram of ISP-NMs and application for cancer treatment. **(B)** Fluorescent intensity of cancer cells after treatment. **(C)** Tumor volume changes during 14 days. *P < 0.05. **P < 0.01. ***P < 0.001 drugs treated groups versus one of control; ^#^*P* < 0.05,^*##*^*P* < 0.01,^*###*^*P* < 0.001 other drugs treated groups versus the group of ISP-NMs+M. Reproduced, with permission, from Zhang et al. (2020a). Copyright 2020, Elsevier.

Ferrite nanoparticles are also used to catalyze the production of molecular oxygen to overcome tumor hypoxia, improving the ROS-mediated tumor therapeutic effect. Hyeon et al. designed manganese ferrite nanoparticle anchored mesoporous silica nanoparticles loaded with molecule chlorin e6 (MFMSNs-Ce6) (Kim et al., 2017). The manganese ferrite could catalyze decomposition of H_2_O_2_ to evolve O_2_, which could be further converted to singlet oxygen by photosensitizer Ce6, improving photodynamic therapeutic outcomes for hypoxic tumor. Some similar studies were carried out by other researchers. Lin et al. prepared photosensitizer-loaded and PEG-modified MnFe_2_O_4_-decorated large-pore mesoporous silica-coated β-NaYF_4_:20%Yb,2%Er@β-NaYF_4_ upconversion nanoparticles (UCMnFe-PS-PEG) as NIR light-mediated and O_2_ self-sufficient photodynamic therapy (PDT) agents (Ding et al., 2019). The sub-10 nm MnFe_2_O_4_ nanoparticles not only provided magnetic guidance to the tumor but also worked as a Fenton catalyst to generate O_2_ *in situ* to overcome tumor hypoxia. The tumor growth was greatly inhibited, and some of the tumors even disappeared after 16 days of treatment. A biocompatible nanoplatform [MnFe_2_O_4_@metal-organic framework (MOF)] developed by Zhang et al. using a coating of porphyrin-based MOF as the photosensitizer and manganese ferrite nanoparticle (MnFe_2_O_4_) as the enzyme can not only catalyze H_2_O_2_ to produce O_2_ to overcome the tumor hypoxia but also consume glutathione, achieving better therapeutic efficacy (Yin et al., 2019). The tumor growth was considerably suppressed after mice were treated with MnFe_2_O_4_@MOF-PEG (i.v. injection of 200 μl, 6.25 mg kg^−1^ TCPP) and laser irradiation (0.8 W cm^−2^, 8 min, 24 h post-i.v. injection). Zhang et al. showed “all in one” theranostic agents copper ferrite nanospheres (CFNs), in which the coupling between Fe^2+^/Fe^3+^ and Cu^+^/Cu^2+^ redox pairs could produce more •*OH* and O_2_ through Fenton reactions under 650-nm laser illumination (Liu et al., 2018). The produced O_2_ could be further converted into $O_{2}^{-}$ by photogenerated electron/hole pair for synergistic tumor ablation of photoenhanced chemodynamic therapy/photodynamic therapy/photothermal therapy. Lin et al. synthesized a novel nanoplatform composed of hollow iron oxide nanoparticles and hematoporphyrin sonosensitizers (HP-HIONs) (Zhang et al., 2020d). The HIONs possessed nanozyme activity for catalyzing decomposition of hydrogen peroxide to produce O_2_, which could be further converted to ROS by sonodynamic therapy for efficient cancer cell apoptosis. Wei et al. synthesized iron oxide nanoparticles-loaded stomatocytes@ZnPc nanomotors (ISP-NMs), in which IONPs catalyzed decomposition of endogenous H_2_O_2_ to generate O_2_ as propelling force to expand the distribution of ZnPc (Zhang et al., 2020a). The generated O_2_ was supplied to produce more ROS (^1^*O*_2_), enhancing PDT performance (Figure 12).

**Figure 13:**
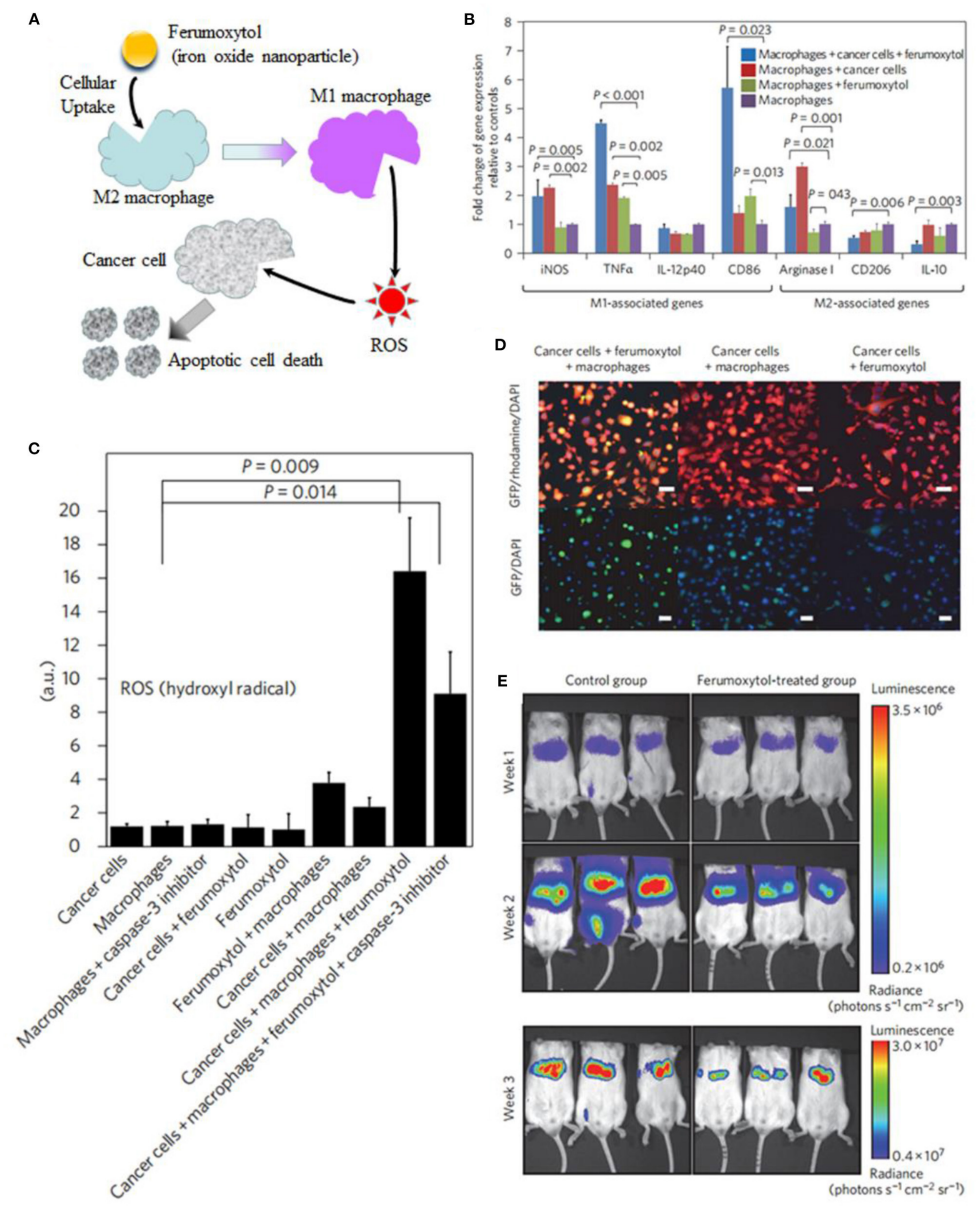
**(A)** Schematic illustration of ferumoxytol-altered polarization of tumor-associated macrophages to release ROS, inducing cell death. **(B)** Signs of proinflammatory macrophage activation. **(C)** Quantitative measures of hydroxyl radical. **(D)** Coculture leads to increased caspase-3 expression of cancer cells. **(E)** Serial bioluminescence imaging after intravenous injection of ferumoxytol at a dose of 10 mg Fe kg^−1^. Reproduced, with permission, from Tarangelo and Dixon (2016) and Zanganeh et al. (2016). Copyright 2016, Macmillan Publishers Limited.

Some other strategies have also been developed to increase ROS generation. Daldrup-Link et al. coincubated adenocarcinoma with iron oxide nanoparticle compound ferumoxytol and macrophages (Zanganeh et al., 2016). Ferumoxytol nanoparticles increased presence of proinflammatory M1 macrophages in the tumor to enhance the production of hydrogen peroxide and hydroxyl radical for macrophage-modulating cancer immunotherapies (Figure 13). Chen et al. constructed double-layered vesicles with Fe_3_O_4_ face-to-face localized in the inner side and Au extended to the outer side by self-assembly of iron oxide–gold Janus nanoparticles (Fe_3_O_4_-Au JNPs) using hydrophilic poly(ethylene glycol)-grafted Au and poly(lipid hydroperoxide)-*co*-poly(4-vinyl pyrene)-coated Fe_3_O_4_. In the acidic tumor environment, the vesicles disassembled into single JNPs, allowing Fe_3_O_4_ to react with H^+^ to release Fe^2+^. The released Fe^2+^ further reacted with poly(lipid hydroperoxide) to generate reactive oxygen species (^1^*O*_2_) and increase intracellular oxidative stress for better inhibition of tumor growth (Song et al., 2019).

## Conclusion and Future Outlook

In the past nearly 10 years, ROS-mediated cancer therapy using ferrite nanoparticles has been rapidly developed, and researchers have published a large number of related publications (summarized in Table 1). This review was carried on the classification and summarization of the application of ferrite nanoparticles in ROS-mediated cancer therapy. Based on the analysis of the current literature, it can be seen that various modification strategies for the ROS-mediated cancer therapies based on ferrite nanoparticles are producing more and more successful results, especially in combination with drugs, biological and chemical agents, and/or co-exposure of other energy fields such as X-rays, lasers, and alternating magnetic fields, becoming potential effective tumor therapy strategies. However, to date, only iron oxide nanoparticles have been approved for the magnetic response diagnosis and the magnetic hyperthermia tumor therapy (Park et al., 2017; Shi et al., 2017). Few clinical trials have been reported for any tumor therapy based on ferrite nanoparticle-induced ROS (Liang et al., 2020). The ferrite nanoparticle-induced ROS can kill the cancer cell and also can trigger the toxic effect on the normal tissues and vasculature. Therefore, in order to solve this dilemma, on the one hand, the high-performance ferrite nanoparticles should be developed to produce much more ROS so that the dosage of the particles can be reduced. On the other hand, the smart (stimulus-responsive) ferrite nanoparticles should be designed to produce more controllable ROS in tumor tissue and little to no ROS outside tumor tissue.

**Table 1:** Summary of current ferrite nanoparticles used for ROS-mediated cancer therapy.

**ROS production**	**Ferrite-based nanoplatform**	**Brief description**	**References**
Intrinsic fenton reaction	Fe_3_O_4_ (6, 13 nm)	Smaller size, higher enzyme activity	Zhang et al., 2013
	Fe_3_O_4_ (6, 9, and 14 nm)	Small size NPs destroy mitochondria, while larger size destroy cytomembrane	Xie et al., 2016
	SPIONs (7.3, 15.1, 30.0 nm)		Zhang et al., 2020c
	FeO_x_-MSNs	pH responsive, delivered to acidic lysosomes	Fu et al., 2015
	Fe_3_O_4_ nanocluster, nanoflower, and nanodiamond	Fe_3_O_4_ nanodiamonds induce the highest cell killing effect	Fu et al., 2017
	CuFe_2_O_4_	Non-ferrous metal species regulate the ROS production	Ahamed et al., 2016
	MB-CuFe NPs		Kuo et al., 2020
	SnFe_2_O_4_		Lee et al., 2017
	Iridium oxide and iron oxide		Shaikh et al., 2020
	CuO, γFe_2_O_3_, CuZnFe_2_O_3_		Siddiqui et al., 2020
	IONPA	Coating reduces nanoparticle size	Thoidingjam and Tiku, 2019
	UC-IONP, CA-IONP, SP-IONP, AS-IONP, DA-IONP	Coatings decreases surface reactivity	Mai and Hilt, 2019
	Fe_2_O_3_@DMSA, Fe_2_O_3_@APTS	DMSA-coating promotes uptake efficiency	Xie et al., 2020
	Fe_3_O_4_/Fe@F-SiO_2_/PDA	Catalase-imprinted shell inhibits catalase activity to elevate H_2_O_2_ level	Chen et al., 2017a
	mag. SLPs	Targeting molecules, responsive molecules, improved delivery efficiency and selectivity	Swietek et al., 2020
	Mito-PANPs		Pandey et al., 2020
	Fe_5_C_2_@Fe_3_O_4_	Gradient core-shell structure, differential release	Yu et al., 2019
	PEGylated FePt-Fe_3_O_4_ + doxorubicin	Combining ferrite nanoparticle and chemotherapeutic drugs, chemical and biological agents, etc. improves ROS-mediated tumor therapy.	Sahu et al., 2015
	H_2_O_2_/Fe_3_O_4_-PLGA polymersome		Li et al., 2016
	Fe_3_O_4_ + (rapamycin or carboplatin)		Kojima et al., 2018
	DOX-ICG@Fe/FeO-PPP-FA nanocapsules		Wang et al., 2019c
	TRAIL/Apo2L-iron oxide nanoparticles		Shi et al., 2020
	*S. aromaticum* + PVP + Fe-ONPs		Thenmozhi, 2020
	Iron oxide nanoparticles	Broad applicability to a wide range of cancers: HepG2, A549, MCF-7, OVCAR-3, SKOV-3, HeLa S3, AGS, metastatic OC, OTSCC, etc.	Ahamed et al., 2013
	Nickel ferrite nanoparticles		Ahamed et al., 2015
	Magnetite iron oxide nanoparticles		Gokduman, 2019
	Fe_3_O_4_@LEC-CUR-PLGA-MMS		Ayyanaar et al., 2020
	Fe_3_O_4_@CPTMOS/TP NPs		Habibzadeh et al., 2020
	α-Fe_2_O_3_		Ramalingam et al., 2020
	SPIONs		Jahanbani et al., 2020
	SPIONs	Mechanisms: mitochondrial electron transport chain, antioxidant-related genes, mTOR-Akt-p70S6 K and ATG7, etc.	He et al., 2016a
	9 nm Fe_3_O_4_ NPs		Ye et al., 2020
	PEI-MNPs		Man et al., 2020
External field enhanced ROS	NiFe_2_O_4_/C	Enhanced by ultrasound	Gorgizadeh et al., 2019
	Citrate-coated SPIONs	Increased ROS production under X-ray irradiation, etc.	Klein et al., 2012
	9–20 nm (γ-Fe_2_O_3_)_1−*x*_(Fe_3_O_4_)_x_		Klein et al., 2014
	Cetuximab-IONPs		Bouras et al., 2015
	TAT-Fe_3_O_4_		Hauser et al., 2016b
	PA-SAM functionalized Fe_3_O_4_ and CoFe_2_O_4_ MNPs		Klein et al., 2018
	Zn^2+^-doped magnetic nanoparticles	Improved catalytic activity under NIR photothermal energy	Qi et al., 2016
	Bacterial magnetic nanoparticles		Chen et al., 2016
	IONPs-ICG-HA		Wang et al., 2019a
	γGDYO-Fe_3_O_4_-CREKA (TTIS)	Nanoplatform depolymerizes under NIR Photothermal energy	Min et al., 2020
	Pt/Fe_3_O_4_@SP-PLGA		You et al., 2019
	FeTiO_3_@Fe_2_O_3_	650 nm laser irradiation formed photoexcited electron–hole	Ou et al., 2020
	TAT-IONP	Improved catalytic activity under AMF magnetic heat	Hauser et al., 2016a
	Doxorubicin-loaded Fe_3_O_4_ nanoparticles		Orel et al., 2018
	mHAP		Yang et al., 2018
	Magnetic hydrogel nanozyme (MHZ)		Wu et al., 2019
	FVIOs-GO-CREKA		Liu et al., 2020
	Iron oxide magnetic nanoparticles	Magnetic heating superior to extrinsic hot air heating	Ludwig et al., 2017
	Co_0.2_Mn_0.8_Fe_2_O_4_	0.5 T static magnetic field	Marycz et al., 2017
	IONF@CuS	Synergistic effect of multi-field coupling (AMF and laser irradiation)	Curcio et al., 2019
	Fe_3_O_4_-Pd		Ma et al., 2019
	Manganese doped-iron oxide nanoclusters (MNCs)		Gupta and Sharma, 2020
Cascades increased ROS	SPION micelles	β-lapachone increases H_2_O_2_	Huang et al., 2013
	LaCIONPs		Wang et al., 2019b
	Fe_3_O_4_@C-FA	Ascorbic acid increases H_2_O_2_	An et al., 2013
	Vitamin C-conjugated Fe_3_O_4_		Pal and Jana, 2020
	FePt-NP2	Cisplatin activates NADPH oxidase to generate H_2_O_2_	Ma et al., 2017
	FeGd-HN@Pt@LF/RGD2		Shen et al., 2018
	FA/Pt+si-GPX4@IONPs		Zhang et al., 2020b
	GFD NCs	Glucose oxidase consumes glucose to generate H_2_O_2_	Huo et al., 2017
	Fe/G@R-NRs		Ke et al., 2019
	Fe_3_O_4_@PDA/GOx NPs		Zhang et al., 2019
	Nb_2_C-IO-CaO_2_	CaO_2_ as H_2_O_2_ supplier	Gao et al., 2019
	GO-FeO_x_H	Graphene oxide produces ROS under laser irradiation.	He et al., 2017
	MFMSNs-Ce6	Ferrite nanoparticles catalyze decomposition of H_2_O_2_ to O_2_ to overcome tumor hypoxia, improving ROS-mediated cancer therapy.	Kim et al., 2017
	UCMnFe-PS-PEG		Ding et al., 2019
	MnFe_2_O_4_@MOF-PEG		Yin et al., 2019
	Copper ferrite nanospheres (CFNs)		Liu et al., 2018
	HP-HIONs		Zhang et al., 2020d
	ISP-NMs		Zhang et al., 2020a
	Ferumoxytol nanoparticles	Ferumoxytol acted on tumor-associated macrophages to adapt an antitumor “M1” phenotype, enhancing macrophage ROS production.	Zanganeh et al., 2016
	Fe_3_O_4_-Au JNPs self-assembled vesicles	poly(lipid hydroperoxide) reacts with released Fe^2+^ to generate ROS	Song et al., 2019

Having achieved the excellent performance of ROS-mediated cancer therapy based on ferrite nanoparticles on small animal model, there are still many important challenges before clinical application. First, further studies on the development of strategies for controllable synthesis of ferrite nanoparticles in large scale are needed to satisfy the requirement for clinical translation and commercialization. Second, the biosafety should be fully investigated on large animals, as most of the current ferrite nanoparticles biosafety evaluation *in vivo* is based on small animals, and the biosafety of the nanoparticles remains largely unexplored in large animals and even in human models. It is appealing to combine efforts from the researchers in the fields of oncology, biochemistry, nanotechnology, medicine, and materials to shed light on the future of ROS-mediated cancer therapy based on ferrite nanoparticles.

## Author Contributions

SY, SZ, and MZ wrote the manuscript. SY and HZ revised the manuscript. HF provided useful suggestions. All authors contributed to the article and approved the submitted version.

## Conflict of Interest

The authors declare that the research was conducted in the absence of any commercial or financial relationships that could be construed as a potential conflict of interest.
